# Quantification of the Steric Properties of 1,8-Naphthyridine-Based Ligands in Dinuclear Complexes

**DOI:** 10.1021/acs.organomet.2c00458

**Published:** 2022-12-02

**Authors:** Lars Killian, Roel L. M. Bienenmann, Daniël L. J. Broere

**Affiliations:** Organic Chemistry and Catalysis, Institute for Sustainable and Circular Chemistry, Faculty of Science, Utrecht University, Universiteitsweg 99, 3584 CG Utrecht, The Netherlands

## Abstract

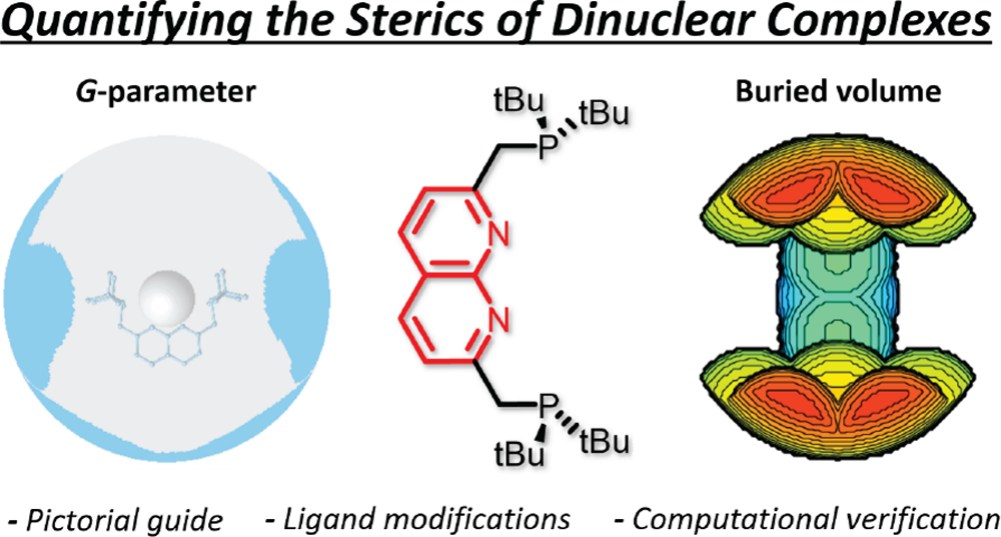

Steric properties of ligands are an important parameter for tuning the reactivity of the corresponding complexes. For various ligands used in mononuclear complexes, methods have been developed to quantify their steric bulk. In this work, we present an expansion of the buried volume and the G-parameter to quantify the steric properties of 1,8-napthyridine-based dinuclear complexes. Using this methodology, we explored the tunability of the steric properties associated with these ligands and complexes.

## Introduction

Steric encumbrance around the metal center of an organometallic complex is an important parameter which can greatly influence the reactivity of such a complex.^1^ This parameter can be tuned by adjusting the ligand design to accommodate for more space to bind additional ligands or less space to prevent extra ligands from binding. Tuning the steric properties of ligands has not only been useful for coordination chemists, but it has also been extensively exploited in homogeneous catalysis. Here, the steric properties of ligands have been used to drive the regio- and enantioselectivity of otherwise aselective reactions.^2−6^

**Figure 1:**
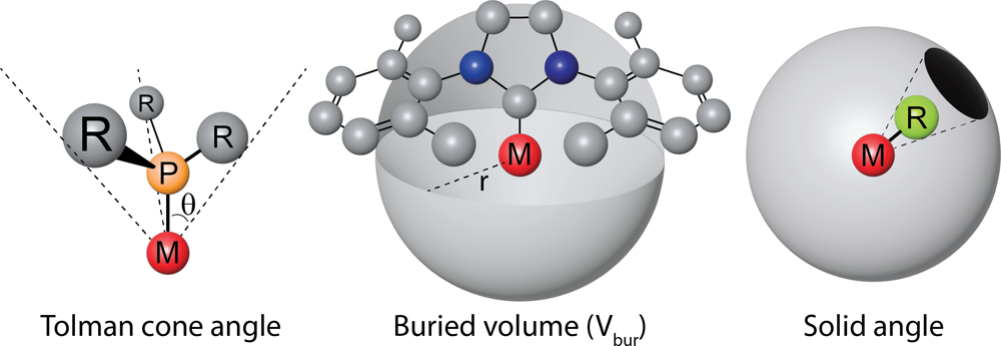
Schematic representation of the established steric parameters. Tolman cone angle (left) where 2θ is the cone angle. Buried volume (middle), the area of the sphere around the metal occupied by the ligand is the buried volume. Solid angle or *G*-parameter (right), where the parameter is the fraction of the sphere in the shadow cast on the sphere by a point light on the metal.

Because of the important role sterics play in determining the reactivity of complexes, understanding and quantifying the steric environment around the metal is important for rational ligand design. The quantification of steric properties in a constructive manner is not trivial because not all bulk on a ligand will influence the metal center in the same way. This has led to different descriptors of steric encumbrance being developed for different types of ligands, starting with the seminal work of Tolman and coworkers who quantified the steric properties of PR_3_ ligands with the Tolman cone angle (Figure 1, left).^7−9^ This parameter measures the angle of the cone formed by the phosphine substituents with the metal atom bound to phosphorus at the top. This steric parameter was shown to correlate well with the substitution equilibria observed for Ni(0)L_4_ (L = PR_3_) complexes.^7−9^ More recently, the Tolman cone angle approach was refined by computing the exact cone angles^10^ or by using the most stable computed conformations of the phosphines, instead of the most folded configuration.^11^ The concept of the Tolman cone angle parameter works effectively for the cone-shaped phosphines, but it does not extend well to other types of ligands which lack the cone shape and symmetry found in tetrahedral phosphines.^12^ Since the seminal work by Tolman, several alternative descriptors of ligand steric strain have been put forward, often with the aim of creating a more general way of measuring steric strain in ligands with increasingly complicated architectures. One of the ways in which such a generalization of steric parameters has been achieved is through the use of solid angles.^13−15^ The solid angle is a geometrical entity (Ω) used in mathematics, which denotes the fraction of the surface of a sphere that is blocked by an object (e.g., a ligand) from a viewpoint (e.g., a metal) in unitless Steradian (sr).^14^ To make this parameter more practical for measuring steric bulk, Guzei and Wendt proposed the *G-*parameter, which is the solid angle expressed as a percentage instead of sr (Figure 1, right).^14^ This method also does not use van der Waals radii for the atom size but rather the atomic zero energy point radii (*R_z_*). In addition to proposing the *G-*parameter, they provided the free solid-*G* program with which this parameter can be easily calculated from atomic coordinates.^16^

Another approach to overcome the limitations of the Tolman cone-angle approach is the buried volume parameter (*V*_bur_) introduced by Nolan and co-workers for quantifying the steric properties of N-heterocyclic carbenes (NHCs).^17^ In this model, a sphere is placed around the metal center and the fraction of the sphere that is occupied by the ligand is calculated (Figure 1, middle).^17,18^ This parameter can be easily calculated using the free *SambVca* 2.1 A web application.^19^ The buried volume approach has been adapted for quantifying the steric parameters of a wider variety of ligands than NHCs alone.^20−23^ For example, this method has been applied to mononucleating pincer complexes by Roddick^22^ and Kamitani et al.^23^ The latter elegantly showed that the buried volume can be used to explain trends in the catalytic hydrosilylation activity of PNN iron pincer complexes with different functional groups on the phosphine.^23^ This shows that the buried volume approach can be used for understanding reaction mechanisms as well as for rationally improving homogeneous catalysts.

Recently, there has been an increasing interest in the investigation of complexes that contain two metal atoms in close proximity.^24−28^ These dinuclear complexes can provide access to distinct reactivity from mononuclear analogues through metal–metal cooperativity (MMC). Despite this, both from a coordination chemistry and from a homogeneous catalysis perspective, these dinuclear complexes are underexplored compared to their mononuclear counterparts. To aid the development of this type of complexes, reliable characterization of the sterics of these complexes would be beneficial. Although there are examples in the literature in which *V*_bur_ calculations are applied to dinuclear complexes, the buried volume maps are only used for visualization of the accessible pocket,^29,30^ or the sterics around a bridging ligand are evaluated.^31^ However, to the best of our knowledge, the *V*_bur_ and *G-*parameter methods have not been used to quantify the sterics of the combined dinuclear binding site. As these methods were developed for mononuclear complexes, it is unclear if they can be reliably expanded to complexes wherein two metals are present at varying distances. The validation of these steric quantification methods for dinuclear complexes provides a tool that enables rational tuning of the steric environment of the dinuclear active site through specific ligand modifications. Additionally, a quantifiable metric for steric encumbrance in these complexes is crucial for data-driven approaches to improve the ligand design.^19^

The 1,8-naphthyridine motif is used in various dinucleating ligands as the positioning of the two nitrogen atoms is ideal to bind two metals in close proximity.^25,32−35^ Combined with the possibility to incorporate additional donor fragments via the 2,7-positions, 1,8-naphthyridines are considered a “privileged” motif for dinucleating ligands.^36^ Herein, we report the systematic quantification of the steric encumbrance of 1,8-naphthyridine-based dinuclear complexes. For quantifying the steric environment of the dinuclear binding site in these ligands, we used the *V*_bur_ and *G*-parameter methods. The effect of the choice in the sphere size and sphere origin is investigated to support a robust methodology for quantifying the steric parameters in various dinuclear systems. Detailed written, pictographic, and videographic tutorials on the application of these methods are provided as Supporting Information. In addition, this methodology is used to investigate the influence of different ligand modifications on the steric encumbrance of dinuclear PNNP complexes developed in our group. Finally, the method is also shown to give a good correlation between the steric encumbrance and the calculated energy for the dimerization of ^R^(PNNP*)Cu_2_H complexes.

## Results and Discussion

### Buried Volume of PNNP Ligands

**Figure 2:**
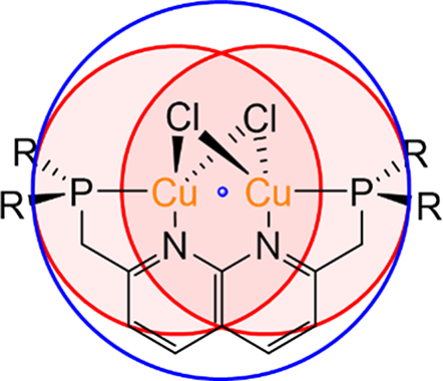
Structure of ^R^PNNPCu_2_Cl_2_, with a schematic drawing of the spheres used for the buried volume analysis (red = Cu centered, blue = origin in the center of the binding pocket).

Buried volume calculations employ a sphere around the metal center and calculate the fraction of the sphere that is occupied by the ligand.^17,18^ To extend this approach to a dinucleating ligand, some standard parameters used in this method have to be adjusted such as the sphere radius and sphere origin. Given that these variables directly influence the calculated buried volume, it is critical to assess their effects. In a dinuclear system, the origin of the sphere can be centered on one of the two metal centers akin to the origin in mononuclear complexes or in the middle between the two metal atoms. The former approach has previously been used for dinuclear cobalt and ruthenium complexes in which the metal centers are not in close proximity.^29,30^ In these reports, the sterics of the full core of the molecule are qualitatively analyzed using *V*_bur_ steric encumbrance maps with a sphere size encompassing the whole molecule. On the other hand, reports on dinuclear 1,8-naphthyridine complexes, in which the metal atoms are in close proximity of each other, have shown that auxiliary ligands or substrates tend to bind in the center between the two metal centers.^25,31,35,37−41^ It therefore reflects the reactivity of these complexes better to choose the center of the binding pocket, in the middle of the two metal atoms, as the origin of the sphere for *V*_bur_ calculations (Figure 2). The standard sphere diameter for mononuclear complexes is 3.5 Å;^42^ however, for a dinuclear binding pocket, the sphere size should be larger to encompass both metals and their surroundings sufficiently. This approach was suggested by the developers of the *SambVca* application used for calculating the buried volumes, however, to our knowledge, it has not been investigated which parameters are appropriate in this case.^42^ Therefore, we started with investigating a suitable sphere size for this approach.

**Figure 3:**
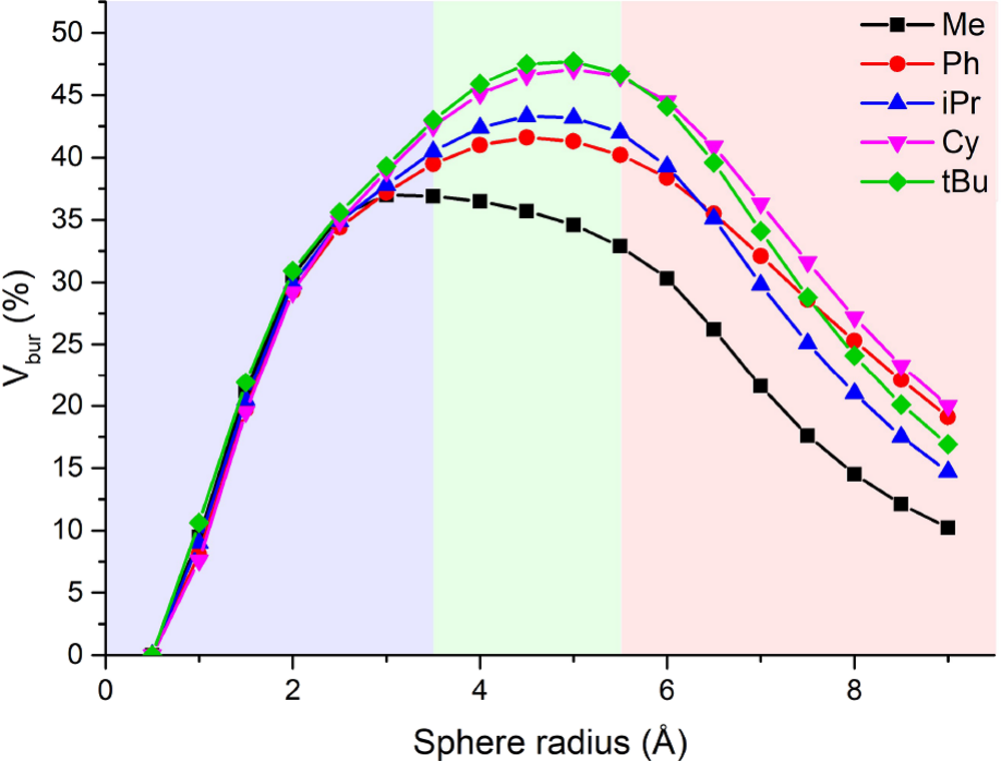
Buried volume of ^R^PNNPCu_2_Cl_2_ (R = Me, Ph, iPr, Cy, or tBu) complexes as a function of the chosen sphere radius. Sphere origin was chosen to be in the center between the two Cu atoms. Blue area indicates that a sphere size is too small, the red area one to large and the green section marks suitable sphere sizes.

If one considers two spheres with a 3.5 Å radius centered on both metal centers in a 1,8-naphthyridine-based complex, which typically display a metal–metal distance of 2–3 Å, a sphere encompassing these two spheres centered at the midpoint would have a radius between 4.5 and 5.0 Å (schematically shown in Figure 2). We therefore expected that a sphere with such a radius centered at the midpoint between the two metal atoms should correlate well with the established 3.5 Å “monometallic” spheres. To evaluate the effect of the sphere size on the calculated buried volume, we calculated the buried volumes of ^R^(PNNP)Cu_2_Cl_2_ (R = Me, Ph, iPr, Cy, or tBu) complexes (Figure 2) using different sphere radii (Figure 3), inspired by the work of Kamitani et al.^23^

The geometries of the ^R^(PNNP)Cu_2_Cl_2_ complexes were optimized with DFT (BP86-D3BJ/def2-TZVP level of theory), and their corresponding buried volumes were calculated using the *SambVca 2.1* application.^19^ The Cu–Cu distances in these optimized geometries range from 2.53 to 2.57 Å, which is within expectations. The complex with *tert*-butyl groups on the phosphorus atoms has been synthesized in our group, and for this complex, the computed geometry was compared to the crystallographically determined structure (Figure S1).^35^ When plotting the buried volume of these complexes against the sphere size, three regimes can be discerned (Figure 3). In the first regime with a small sphere size (<3.5 Å, blue), the buried volume hardly differs between the different ligands. This regime is not useful to calculate the steric encumbrance of dinuclear complexes because the sphere is too small to encompass enough of the ligand to differentiate between the different substituents. In the middle regime (between 3.5 and 5.5 Å, green), there is a difference between the substituents which is illustrative of the steric environment in the core of the complex. When further increasing the sphere size, the order of the buried volume of the different substituents changes. This marks the regime wherein the sphere is too large (>5.5 Å, red) and encompasses most of the ligand, and the trend of the buried volume parameter scales trivially with the size of the substituent. The middle regime between 3.5 and 5.5 Å is most informative of the steric encumbrance of the core of the metal complex. This agrees with the expected sphere radius of ∼5 Å based on a dinuclear system with a M–M separation of ∼3 Å. We will therefore use a sphere size of 5 Å for buried volume calculations from hereon, unless mentioned otherwise.

The obtained buried volumes with a 5 Å sphere in the center of the metal centers (Figure 2, blue) were compared with the values obtained with a 3.5 Å sphere centered at the copper nuclei (Figure 2, red). This showed that the values obtained with both approaches correlate well, which indicates the expansion of this method to dinuclear system works (Figure S4).

### PNNP *G-*Parameter

**Figure 4:**
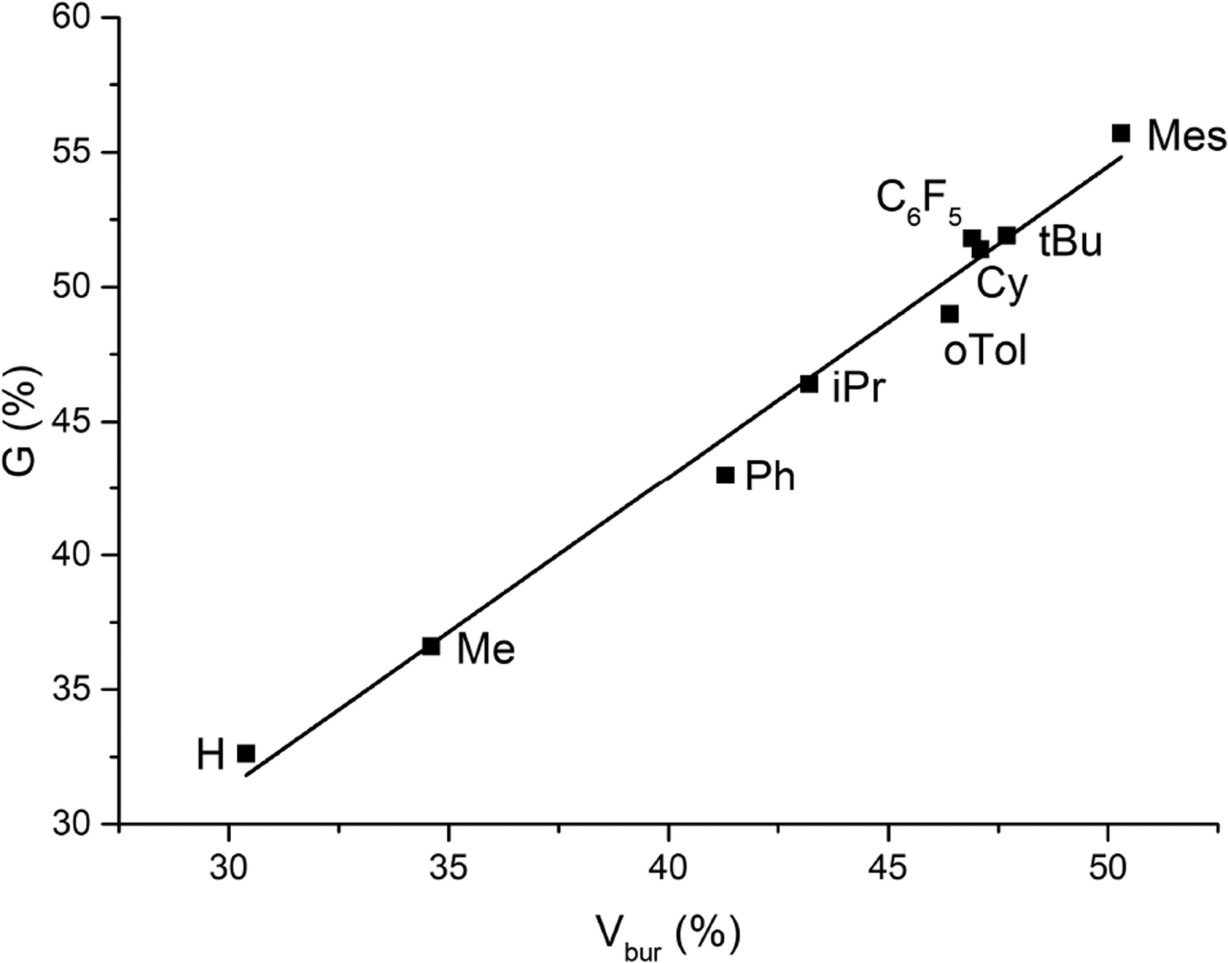
Correlation between the G-parameter and the buried volumes calculated for ^R^(PNNP)Cu_2_Cl_2_ complexes (R = H, Me, Ph, iPr, oTol, Cy, C_5_F_6_, tBu, and Mes).

Next, we were interested to see how robust these results were with respect to the method used to calculate them. Therefore, we also probed the sterics of the previously used series of ^R^(PNNP)Cu_2_Cl_2_ complexes, using the *G-*parameter which was calculated using the solid-G program.^16,14^ To obtain the *G-*parameter, the fraction the surface of a sphere around the molecule that is shielded by the ligand as viewed from the center is calculated.^14^ For mononuclear complexes, this center is the metal atom. For dinuclear complexes, however, the center between the two metal atoms was chosen as the origin of the sphere, for the same reasons as we discussed above for the dinuclear *V*_bur_ calculations. Similarly, this choice was verified by comparing the values obtained with the origin in the middle of the dinuclear binding pocket with those obtained with the origin of the sphere located on one of the metal atoms (Figure S5). The *G-*parameter approach was compared to the buried volume for the original range of complexes, supplemented with R = H, *o*-tolyl, C_6_F_5_ and mesityl (Figure 4), which showed that the calculated values for both correlate well with each other. This indicates that the obtained results for steric encumbrance are robust with respect to the used method.

### Exploring the Sterics of PNNP Complexes

With the established methods for quantifying the sterics of 1,8-naphthyridine complexes in hand, we sought to explore the influence of different ligand modifications on the steric properties of expanded pincer complexes. In this, we focused on PNNP type ligands of which several have been reported.^34,35,43,44^ These insights could help in selecting rational ligand modifications to alter the steric properties in the corresponding complexes.

The previously employed structures of different ^R^(PNNP)Cu_2_Cl_2_ complexes were used in order to investigate over which range of steric demands these expanded pincer ligands could be modified by changing the phosphine substituents. The range in *V*_bur_ and *G* points toward a good degree of tunability of the steric environment of the expanded pincer ligand by changes in phosphine substituents (∼20% difference between R = H and R = Mes, Figure 4).

### Orientational Steric Analysis

**Figure 5:**
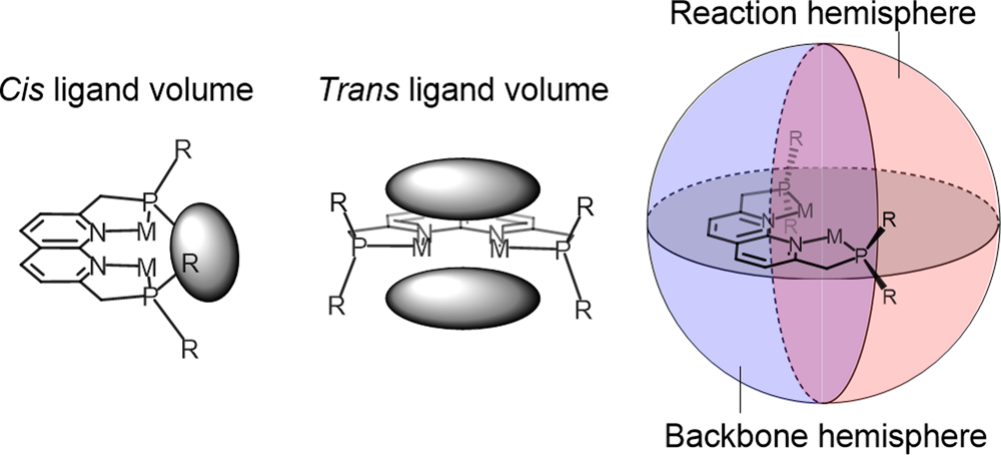
Different ways of characterizing the asymmetry in void space (gray ellipsoids) in “expanded pincer” complexes.

In pincer-type complexes, the ligand “shields” one side of the metal center, due to which the reactivity of these metals typically takes place on the opposite site. To account for this when calculating the steric encumbrance, Roddick described the void spaces around the metal core in pincer complexes in terms of *trans* and *cis* ligand void space (Figure 5).^22^ This classification can be useful when a specific approach or coordination mode of substrates is considered. However, neither the buried volume approach nor the solid angle approach give a direct numerical description of the extent of these void spaces. They can only be inspected visually using the steric map or sphere projections provided by the *SambVca* and *Solid-G* applications.^14,19^ Alternatively, Kamitani et al.^23^ divided the catalytic pocket of iron PNN-type pincer ligands into two hemispheres, one on the side of the ligand backbone and one on the side of the substrate binding pocket. This approach benefits from the ease in which the respective hemispheres can be defined, providing access to a quantifiable steric encumbrance parameter of both, using the buried volume approach. This approach can also be applied to the expanded pincer system (Figures 5 and 11), and both the reaction and backbone hemisphere buried volumes have been calculated for the compounds presented in this work. It is important to note that this hemisphere approach can provide useful insights if a reaction of interest indeed takes place in the reaction hemisphere. If a reaction also involves part of the backbone hemisphere, for example, a metal–ligand cooperative bond activation, the normal buried volume can be more informative.

**Figure 6:**
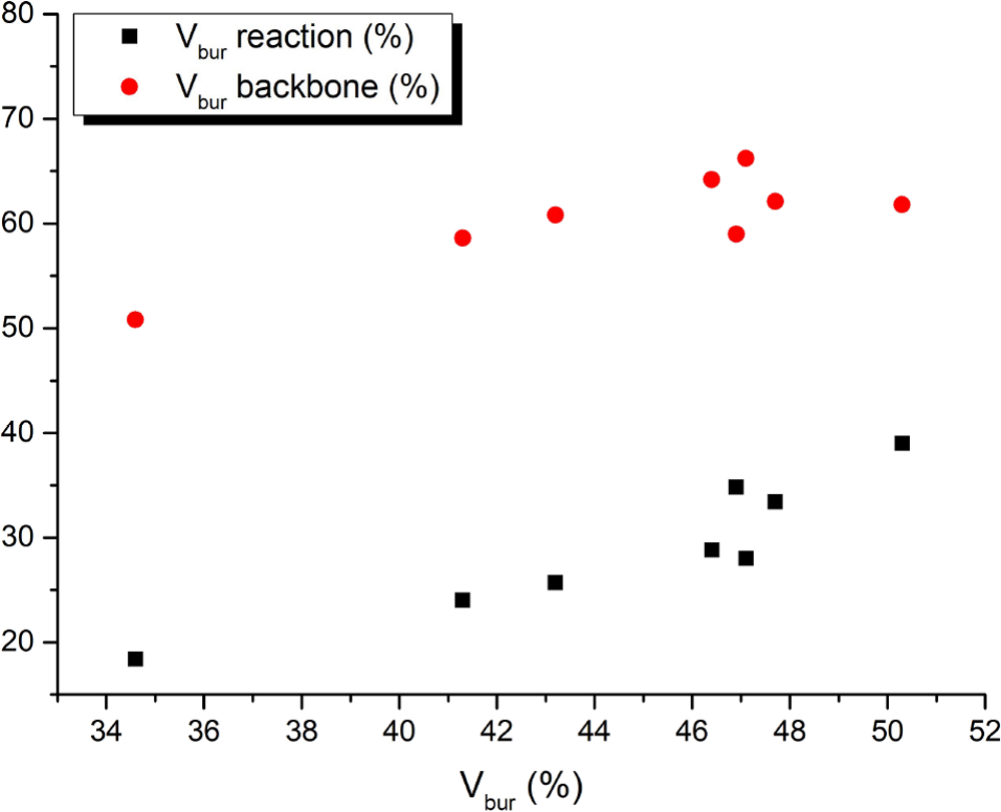
Hemisphere buried volumes for the DFT-optimized geometries of ^R^(PNNP)Cu_2_Cl_2_ complexes plotted against their total buried volume.

In addition to the regular buried volume described earlier, also the hemisphere analysis was performed for the buried volume calculations on the series of ^R^(PNNP)Cu_2_Cl_2_ complexes (Figure 6). The trends in the reaction hemisphere and the backbone hemisphere buried volume do seemingly not correlate well with the total *V*_bur_. Examination of the structures, however, reveals that this is in essence an expression of the different conformations of the ligands. For example, the relatively large backbone buried volume for the ^*o*Tol^(PNNP)Cu_2_Cl_2_ complex is explained by its geometry, in which two of the *o*-tolyl groups are twisted to the backbone (Figure S6). However, the rotation of these *o*-tolyl groups to the front of the molecule might have a small energy barrier and could happen facilely at room temperature. Careful analyses of the structure of the complexes are therefore necessary before drawing strong conclusions based on these hemisphere analyses.

### Backbone Modification Effects

Another feature which is expected to have an influence on the steric congestion around the binding pocket in expanded pincer systems, is the backbone architecture. The backbone of PNNP-expanded pincer complexes can be modified by adjusting the methylene linkers. They can, for example, be changed into heteroatoms such as oxygen to form PONNOP complexes.^34,44^ For mononuclear complexes, the influence of such heteroatoms has been described before.^22^ Alternatively, the methylene linkers in the PNNP ligand can be deprotonated, which affects the rigidity of the ligand and the geometry in related complexes.^35,38^ In addition to synthesizing complexes with such a deprotonated backbone, deprotonation can also occur during a reaction step in a catalytic cycle. The influence of variations in the compositions of the side arms in mononuclear pincer complexes has been described before by Roddick, who showed that the preferred angle of the linkers can influence the sterics.^22^ To investigate the effect of changes in the composition of the side arms in expanded pincer complexes, the steric parameters of a set of ^R^(PONNOP)Cu_2_Cl_2_ complexes was compared to those of the corresponding ^R^(PNNP)Cu_2_Cl_2_ complexes (Table 1).

**Table 1:** Steric Parameters for Different ^R^(PNNP)Cu_2_Cl_2_ and ^R^(PONNOP)Cu_2_Cl_2_ Complexes

compound	*V*_bur_ (%)	*V*_bur_ *r* x *n* (%)	*V*_bur_ backbone (%)	*G* (%)
^iPr^(PNNP)Cu_2_Cl_2_	43.2	24.8	60.8	46.4
^iPr^(PONNOP)Cu_2_Cl_2_	41.8	22.3	61.3	45.9
^*t*Bu^(PNNP)Cu_2_Cl_2_	47.7	33.4	62.1	51.9
^*t*Bu^(PONNOP)Cu_2_Cl_2_	45.5	27.6	63.4	49.8
^Ph^(PNNP)Cu_2_Cl_2_	41.3	24.0	58.6	43.0
^Ph^(PONNOP)Cu_2_Cl_2_	39.5	24.1	54.9	42.1

In all three cases (R = iPr, *t*Bu, Ph), the PONNOP complex shows somewhat (∼2%) less steric bulk than the analogous PNNP complex, both for the *V*_bur_ and *G-*parameters. The cause of this trend in the overall sterics is likely explained by the shorter C–O and P–O bonds, compared to C–C and P–C bonds. This effectively “pulls back” the phosphine groups, reducing steric pressure around the dinuclear binding site. This effect is also to some extent reflected in the hemisphere analysis, in which the *V*_bur_ backbone increases and the *V*_bur_ reaction decreases. The same pull-back effect was also reported for mononuclear pincer complexes.^22^

**Scheme 1:**
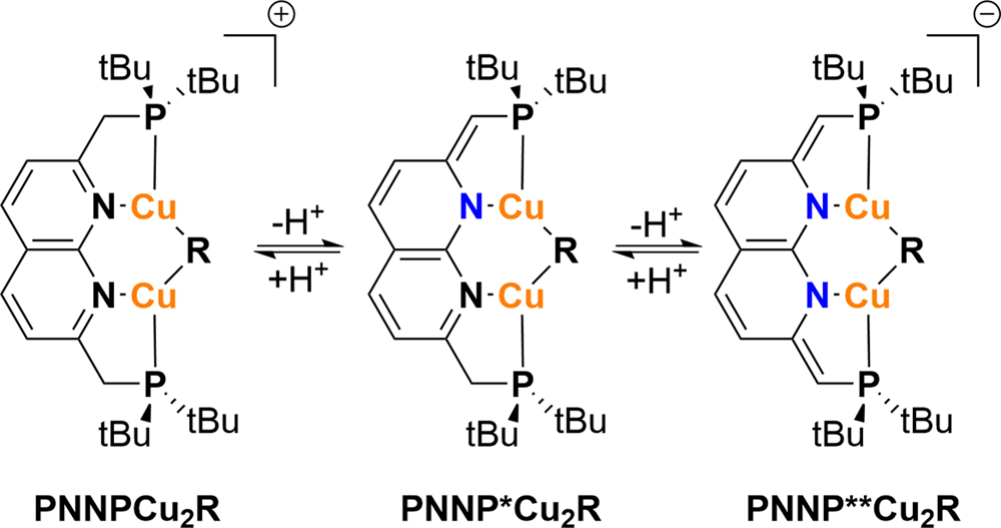
Partial and Full Dearomatization of ^*t*Bu^(PNNP)Cu_2_R Complexes (R = OtBu or Mes)^35,38^

To further assess the influence of changes in the backbone of the expanded pincer ligand on the steric congestion around the catalytic pocket, we investigated the influence of the protonation state of the ligand. To this end, the sterics of a series of fully aromatized, partly dearomatized, and fully dearomatized (Scheme 1, left to right) ^*t*Bu^(PNNP)Cu_2_Mes and ^*t*Bu^(PNNP)Cu_2_O^*t*^Bu complexes was analyzed.^35,38^ These complexes were selected because for these, both experimental and computational data are available. In addition, the Mes and OtBu co-ligands lead to a large variation in the Cu–Cu distance because they are representative for a range of different metal–metal distances.

We anticipated that the smaller C–P bond lengths in the side arms of the anionic ligand or the higher rigidity of those deprotonated linkers might lead to differences in the steric environment of the catalytic pocket. However, the change in the protonation state only leads to a minor change (∼1%) in *V*_bur_ and *G*, as well as the hemisphere analysis when the auxiliary ligand is kept the same. We hypothesize that there are multiple effects on the steric environment upon deprotonation, which cancel each other out. For example, the dearomatized naphthyridine backbone in the PNNP** ligand features smaller C–P distances, thereby “pulling back” the ligand. However, the simultaneous contraction of the Cu–N bonds offsets the expected larger void space. Additionally, the **PNNP** and PNNP* ligands are more flexible and can adopt more bent/twisted configurations which could further influence the steric encumbrance.

**Figure 7:**
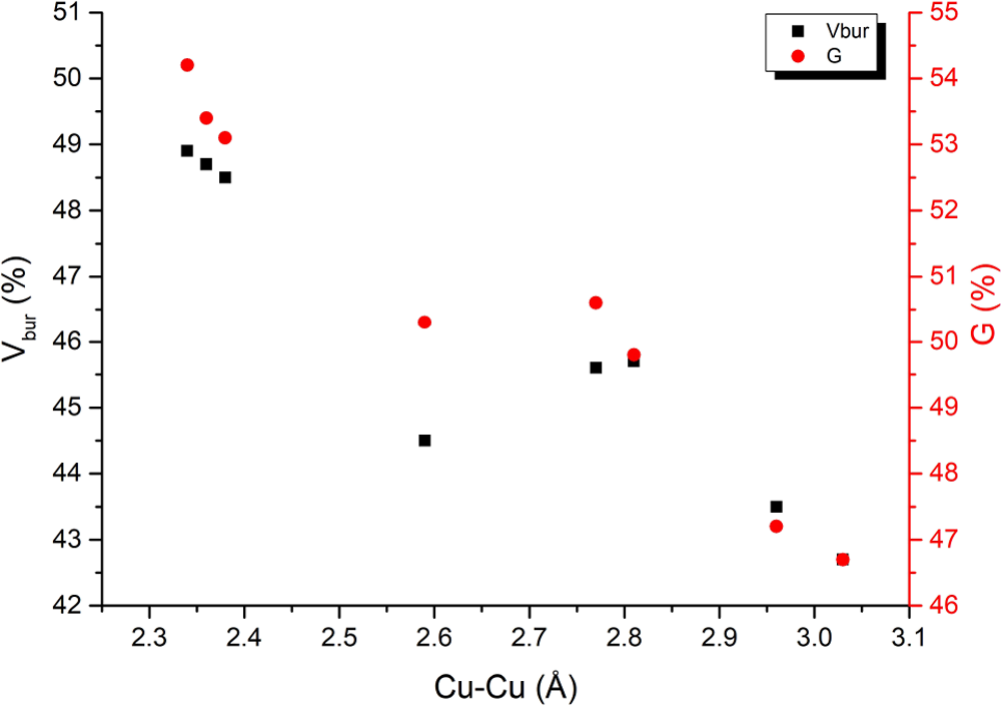
Correlation between the Cu–Cu distance and *V*_bur_ (black) and G (red) for the structures shown in Table 2.

Between different auxiliary ligands (i.e., OtBu or Mes), there is a larger spread in steric parameters *V*_bur_ (42.7–48.9%) and *G* (46.7–54.2%). To probe the origin of this, *V*_bur_ and *G* results from Table 2 are compared with the Cu–Cu distance (Figure 7) as well as the P–P distance (Table S3) of the corresponding complexes. These parameters correlate well with each other. This shows that the metal–metal distance, which is influenced by the auxiliary ligand, is an important parameter determining the steric encumbrance of the dinuclear active site. It is important to note that this effect is dependent on the flexibility of the ligand; for more rigid ligands such as the NDI system reported by Uyeda and coworkers,^39^ this M–M distance dependence is absent (Table S4).

**Table 2:** Steric Parameters for ^*t*Bu^(PNNP) Complexes in Various Protonation States

compound	*V*_bur_ (%)	*V*_bur_ *r* × *n* (%)	*V*_bur_ backbone (%)	*G* (%)	Cu–Cu distance (Å)
^*t*Bu^(PNNP)Cu_2_O^*t*^Bu	45.7	28.9	62.7	49.8	2.81
^*t*Bu^(PNNP)*Cu_2_O^*t*^Bu^a^^,^^b^	42.7	25.0	60.5	46.7	3.03
^*t*Bu^(PNNP)*Cu_2_O^*t*^Bu	45.6	29.4	62.1	50.6	2.77
^*t*Bu^(PNNP)**Cu_2_O^*t*^Bu^a^	43.5	27.2	59.9	47.2	2.96
^*t*Bu^(PNNP)**Cu_2_O^*t*^Bu	44.5	28.6	60.3	50.3	2.59
^*t*Bu^(PNNP)Cu_2_Mes	48.5	34.7	62.2	52.7	2.38
^*t*Bu^(PNNP)*Cu_2_Mes	48.7	35.2	62.2	53.5	2.36
^*t*Bu^(PNNP)**Cu_2_Mes	48.9	36.0	61.9	54.2	2.34

a Reported crystal structure was used.^35^

b The average value for both molecules in the asymmetric unit cell was taken.

Considering the dependence of the steric parameters of PNNP ligands on the M–M distance, it may seem intuitive to consider changing the sphere size in the *V*_bur_ calculations depending on the M–M distance. Doing so does, however, not influence the *V*_bur_ substantially within the range in which the M–M distance reasonably varies (Figure S8, detailed discussion in the SI). This indicates that the use of a 5 Å sphere is a robust choice regardless of variations in the M–M distance.

**Scheme 2:**
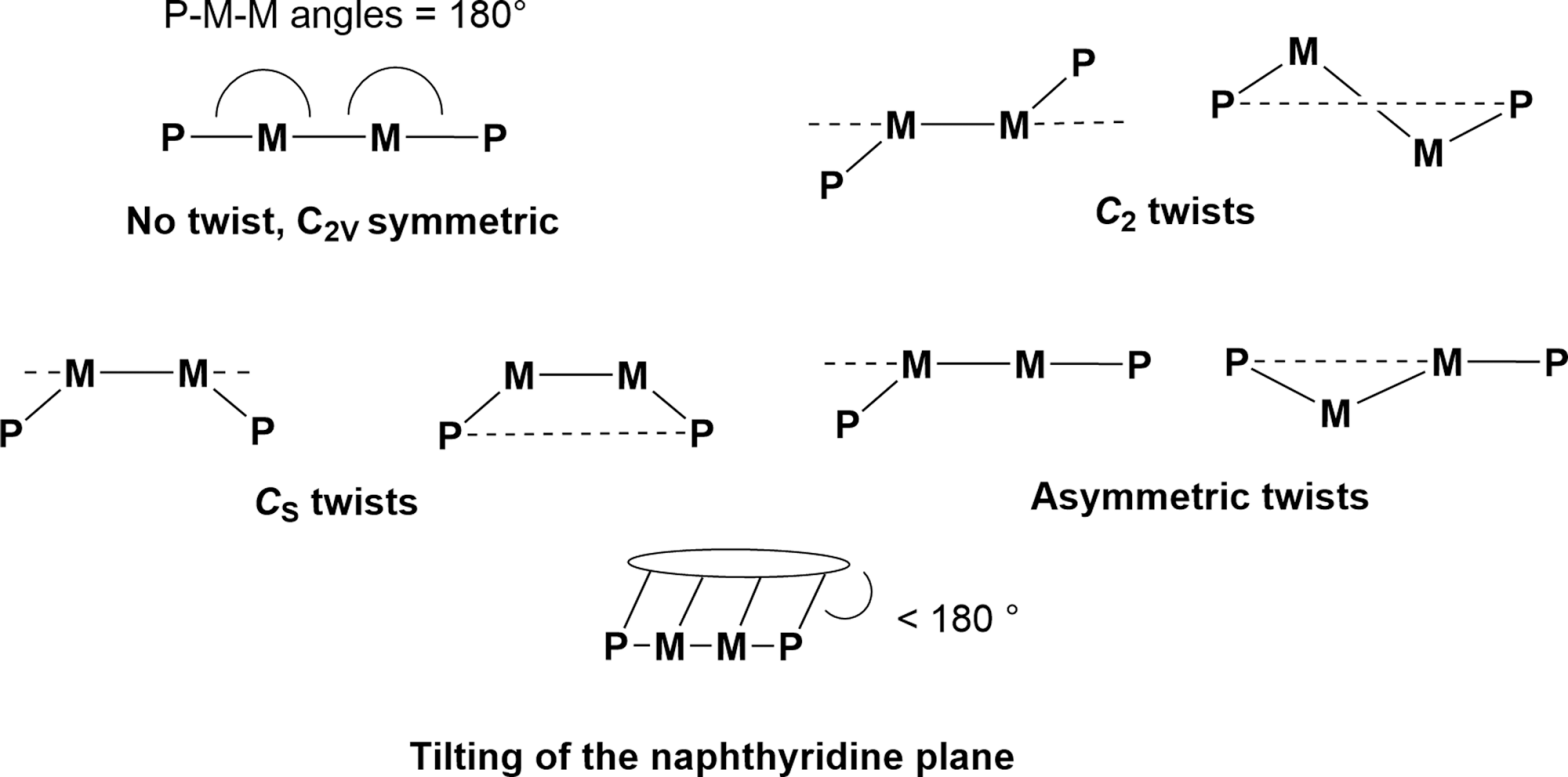
Different Twists and Tilts Observed in Expanded Pincer Ligands The dotted line or ellipse represent the plane of the 1,8-naphthyridine ligand backbone.

When discussing the influences of different ligand modifications on the steric parameters of dinuclear metal complexes, it should be noted that the symmetry of the complex can also influence the sterics. Because the calculations of the steric parameters require either solid state or calculated structures, it is important to consider that these are not always perfectly representative of the geometry in solution. This can, for example, be due to packing effects or small energy barriers for rearrangements (e.g., rotation around a C–P bond). Therefore, it is important to explore the influence of these deviations from the expected symmetry on the calculated steric parameters. For mononuclear pincer complexes, Roddick classified the possible geometries as C_2_, C_s_ and asymmetric twists, depending on the resulting symmetry displayed by the ligand (C_2v_ in the case of no twist).^22^ Parallel observations can be made when considering the conformations of the expanded pincer system. Schematic examples of the different twists and tilts observed in expanded pincer complexes are shown in Scheme 2. Many examples of the calculated and crystallographically determined structures of PNNP complexes display a geometry that is somewhere in between those shown in Scheme 2 (see Figure S9 and references for examples).^35,38^

To assess the influence of the various ligand binding geometries on the steric parameters, the buried volume of ^*t*Bu^PNNPCu_2_Cl_2_ was calculated in the various binding modes depicted in Scheme 2 (Table S6). For these calculations, we used coordinates of previously found structures of ^R^PNNPCu_2_Cl_2_ in which such twists and tilts were observed and replaced the R groups with tBu. The tBu groups were optimized while the coordinates of the metal centers and the rest of the ligand were fixed. This showed that these different geometries lead to a variation of ∼1% in *V*_bur_ and G. For the hemisphere analysis, the deviation is larger (∼4%). This larger difference is due to small and facile rotations of the phosphine substituents which can move them from the reaction to the backbone hemisphere and vice versa. In solution, molecules are dynamic and the small energy barriers associated with such bond rotations are easily overcome. Therefore, these deviations in the steric parameters due to facile rotations should be taken into consideration for flexible ligand systems.

**Figure 8:**
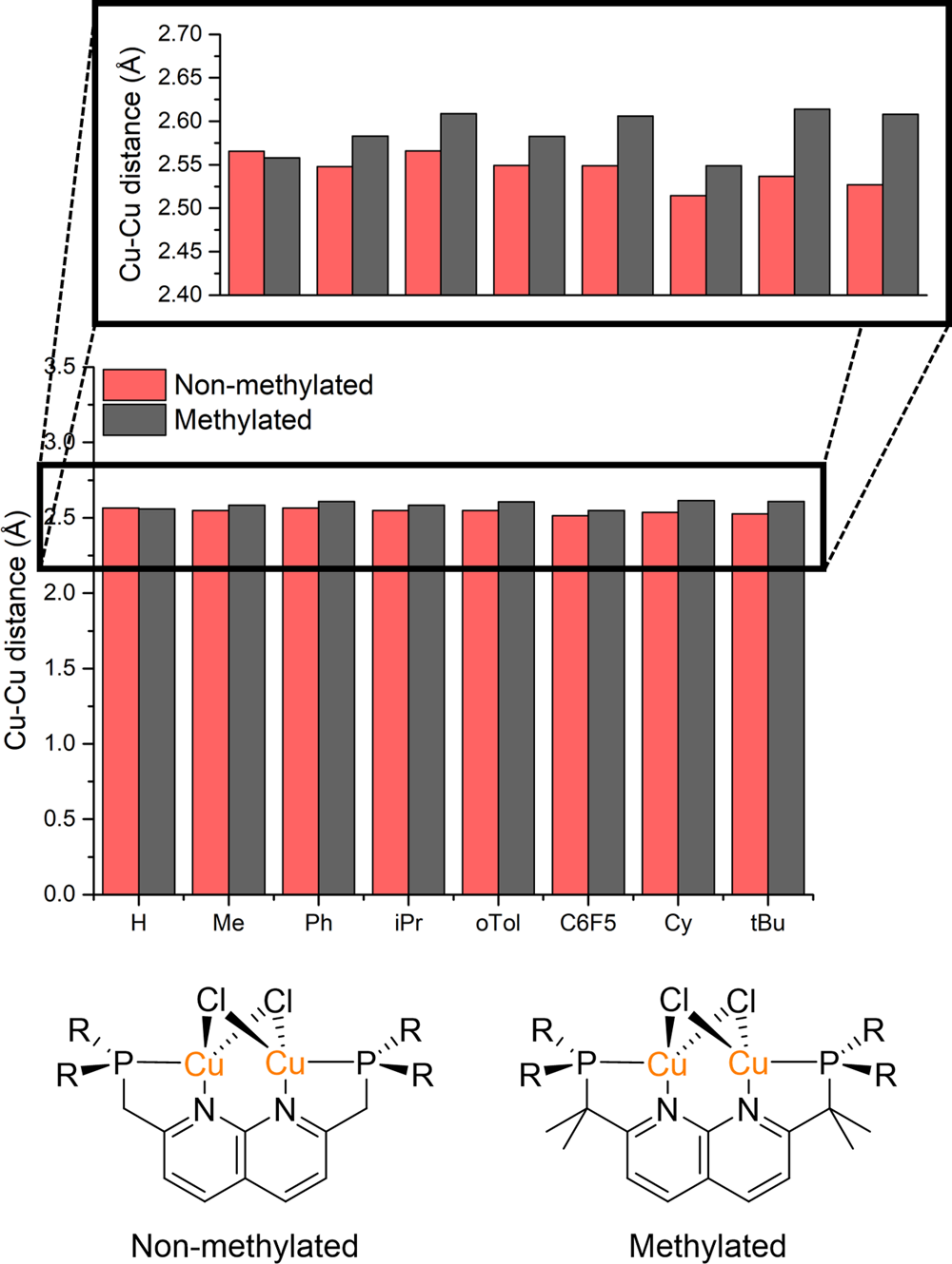
Comparison of the Cu-Cu distance in the ^R^PNNP ligand with and without methylated methylene linkers.

Recently, mononuclear PNP pincer ligands have been modified by the methylation of the backbone to suppress the reactivity (i.e., protonation and deprotonation) of these positions.^45^ An analogous ligand modification can be envisioned for the PNNP expanded pincer ligands (Figure 8), which inspired us to also investigate the sterics of this type of ligand modification. Initially, we hypothesized that adding methyl groups on the methylene linkers would increase the steric demand there, and hence increase the steric congestion by decreasing the Cu–Cu distance (Thorphe-Ingold effect).^46,47^ However, we found that the Cu–Cu distance of the optimized structures with the methylated backbone increased for all the substituents except H (Figure 8). When the optimized structures of the methylated and nonmethylated complexes are compared, it stands out that the methylated complexes are more twisted/tilted (Figures S7–S14). This showcases the important role that different conformations of the ligand can play in determining the steric encumbrance of the binding site in these ligands. We reason that the methyl groups induce these extra twists because a more twisted structure releases steric strain between the methyl groups and the groups on the phosphines. In these twisted structures (except in the case of Me), the distance between the phosphines increases, and with that, the Cu–Cu distance increases. In the case of H as a phosphine substituent, the methyl groups on the backbone are too far away from the H groups to experience steric repulsion, and this likely explains that this Cu–Cu elongation is not observed for this structure. The effect of this increase in the Cu–Cu distance on the buried volume is not easily extracted because the 5 Å sphere for the buried volume also encompasses the additional methyl groups and therefore *V*_bur_ poorly reflects the change in the steric environment in the Cu–Cu core.

**Figure 9:**
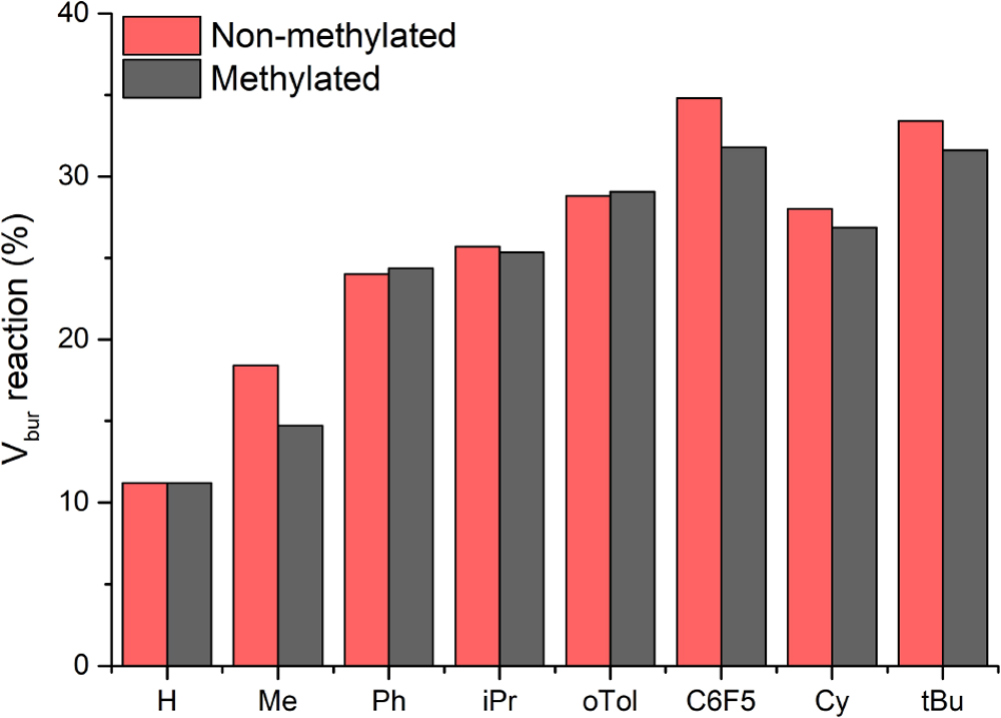
Reaction buried volume of the nonmethylated and methylated ^R^PNNPCu_2_Cl_2_ complexes.

Analogously, the *G-*parameter also takes the methyl groups into account and hence provides an inaccurate comparison of the encumbrance. Therefore, the reaction buried volume was used as a metric for the change in steric encumbrance in the core as this hemisphere does not overlap with the methylene linkers. The comparison of the reaction buried volume between the methylated and nonmethylated ligand indicates that in most cases, the steric encumbrance decreases upon methylation (Figure 9), as expected based on the increased Cu–Cu distance in these cases. For some of the substituents (Ph, oTol, and Mes), a small increase in reaction *V*_bur_ is observed. This seems to be due to minor rotations around the C–P bonds, which cause the substituents on the phosphines to lie for a larger part inside the reaction hemisphere in the case of the methylated structure compared to the nonmethylated one. Visually, these rotations seem facile, hence, we postulate that this is not an effect of methylation, but of the static geometry as discussed before. These results indicate that providing a driving force for inducing a twist or tilt in the complex can be used to alter the steric properties around the metal centers. Therefore, the flexibility of these PNNP ligands is an important factor to consider in the design of complexes featuring such ligands.

### Applicability on Different Naphthyridine Ligands

**Figure 10:**
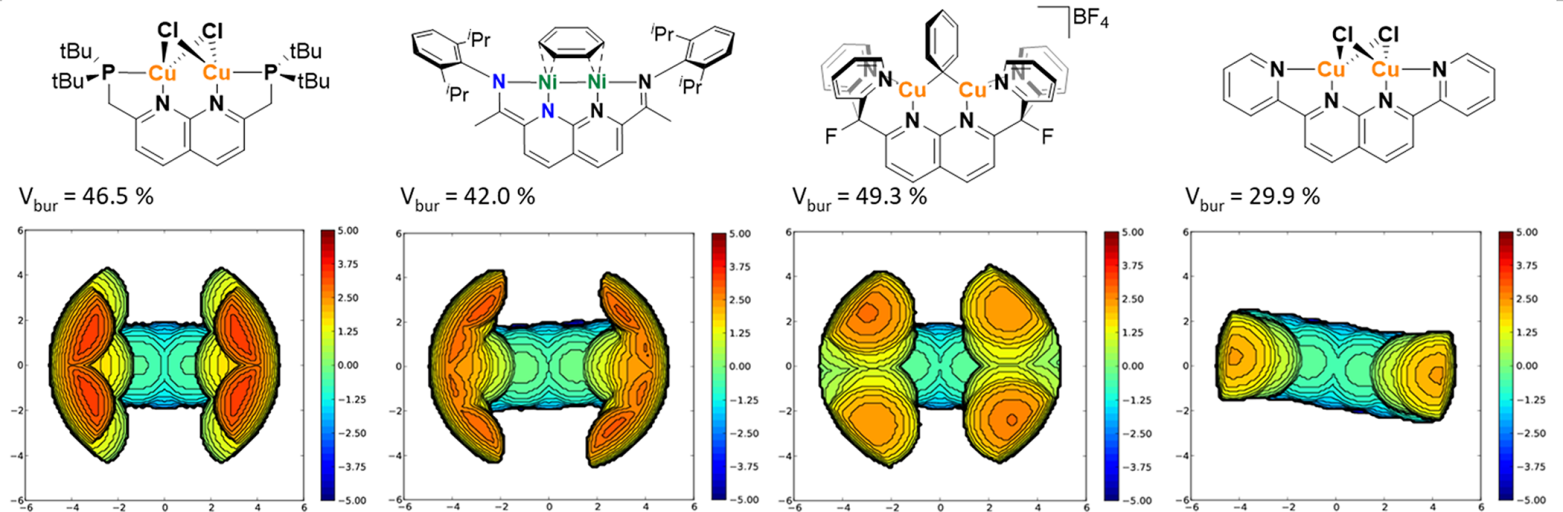
Steric maps of four different 1,8-naphthyridine-based systems and their buried volumes. From the left to right, single-crystal XRD structures have been taken from Broere co-workers,^35^ Uyeda co-workers,^32^ Tilley co-workers,^48^ and Liu co-workers^49^

In order to place the steric environment of the PNNP ligand in the context of other 1,8-naphthyridine-based ligands, the buried volumes of three of these systems and their steric maps were calculated based on the reported X-ray structures (Figure 10). Of the selected examples, the 2,7-bis(fluoro-di(2-pyridyl)methyl)-1,8-naphthyridine system reported by Tilley co-workers^48^ shows the highest buried volume (49.3% for [**L**Cu_2_Mes]BPh_4_), comparable to ^*t*Bu^(PNNP)Cu_2_Mes (49.1%). The ^iPr^NDI ligand reported by Uyeda co-workers^32^ has a buried volume of 42.0% for the investigated dinickel compound. This is comparable to ^*t*Bu^(PNNP)*Cu_2_O^*t*^Bu (42.7%), for example. The planar 2,7-bis(2-pyridyl)-1,8-naphthyridine ligand as reported in a dicopper dichloride complex by Liu et al.^49^ has very little steric congestion around the catalytic pocket (*V*_bur_ = 29.9%), which is lower than even the smallest buried volume calculated for the expanded pincer system in the ^H^(PNNP)Cu_2_Cl_2_ compound (30.4%). The hemisphere analysis and the *G*-parameter follow the trends as expected (Table S7), with one exception. In the complex of Uyeda and coworkers, the G parameter (51.1%) is almost the same as the ones of the complex from Tilley et al. (51.5%) and of the ^*t*Bu^(PNNP)Cu_2_Cl_2_ complex^50^ (46.5%). This stands out because the buried volume of that complex is much lower than that of the other two. The higher G value in this case can likely be attributed to the diisopropylphenyl rings which are perpendicular to the Ni–Ni line. This ring is therefore visible in the *G-*parameter, but it falls largely out of the sphere in the buried volume analysis, hence the discrepancy between the methods. This result indicates that it is important to check which method for the determination of the steric encumbrance is the most suitable for the specific type of complex, especially for comparing different types of ligands with each other. Moreover, the wide range of steric properties observed here for 1,8-naphthyridine-based ligands is important to take into account when comparing reactivity between these complexes in addition to electronic considerations.

### Computational Verification

**Scheme 3:**
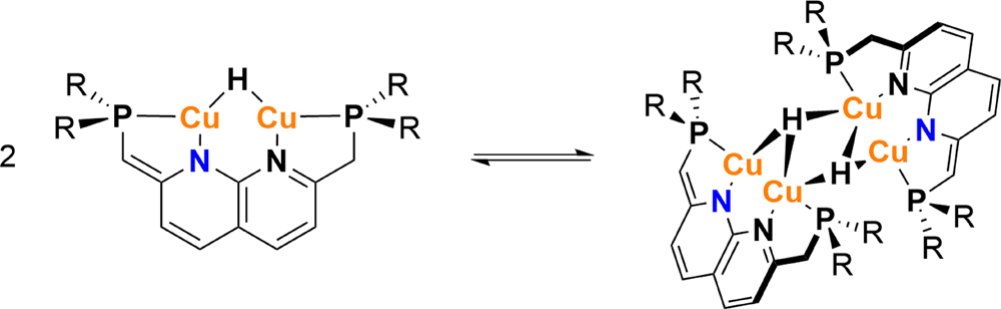
Dimerization Equilibrium between ^*t*Bu^PNNP*Cu_2_H and [^*t*Bu^PNNP*Cu_2_H]_2_

Thus far, we demonstrated that the calculated steric parameters correspond well to the expectations and that they are robust to the method used (i.e., *V*_bur_ or *G*). However, it is important to verify that this also reflects the reactivity of these molecules. Previously, it was reported that ^*t*Bu^(PNNP*)Cu_2_H dimerizes upon formation (Scheme 3), evidently overcoming steric repulsion by the energy gain of dimerization.^35^ Because of the crowded nature of the resulting dimer ([^*t*Bu^(PNNP*)Cu_2_H]_2_), we hypothesized that the dimerization energy should be dependent on the steric encumbrance and hence on the substituents on the phosphines. Therefore, we calculated the dimerization energies for ^R^(PNNP*)Cu_2_H, in which a larger dimerization energy means that dimerization is less exergonic (or more endogonic). These calculations showed positive dimerization energy of 7.7 kcal/mol for [^*t*Bu^(PNNP*)Cu_2_H]_2_, despite experimental observations showing that it is a dimer in the solution and solid state.^35^ We postulate that this is an error introduced by the lack of dispersion correction in the DFT method because a dispersion correction overestimates the dispersive interaction within such dimeric structures, as was shown before (see the SI for detailed discussion).^35^ Because we are interested in identifying the effect of steric encumbrance on this equilibrium, however, a consistent underestimation of the dispersion energy should not influence the results.

**Figure 11:**
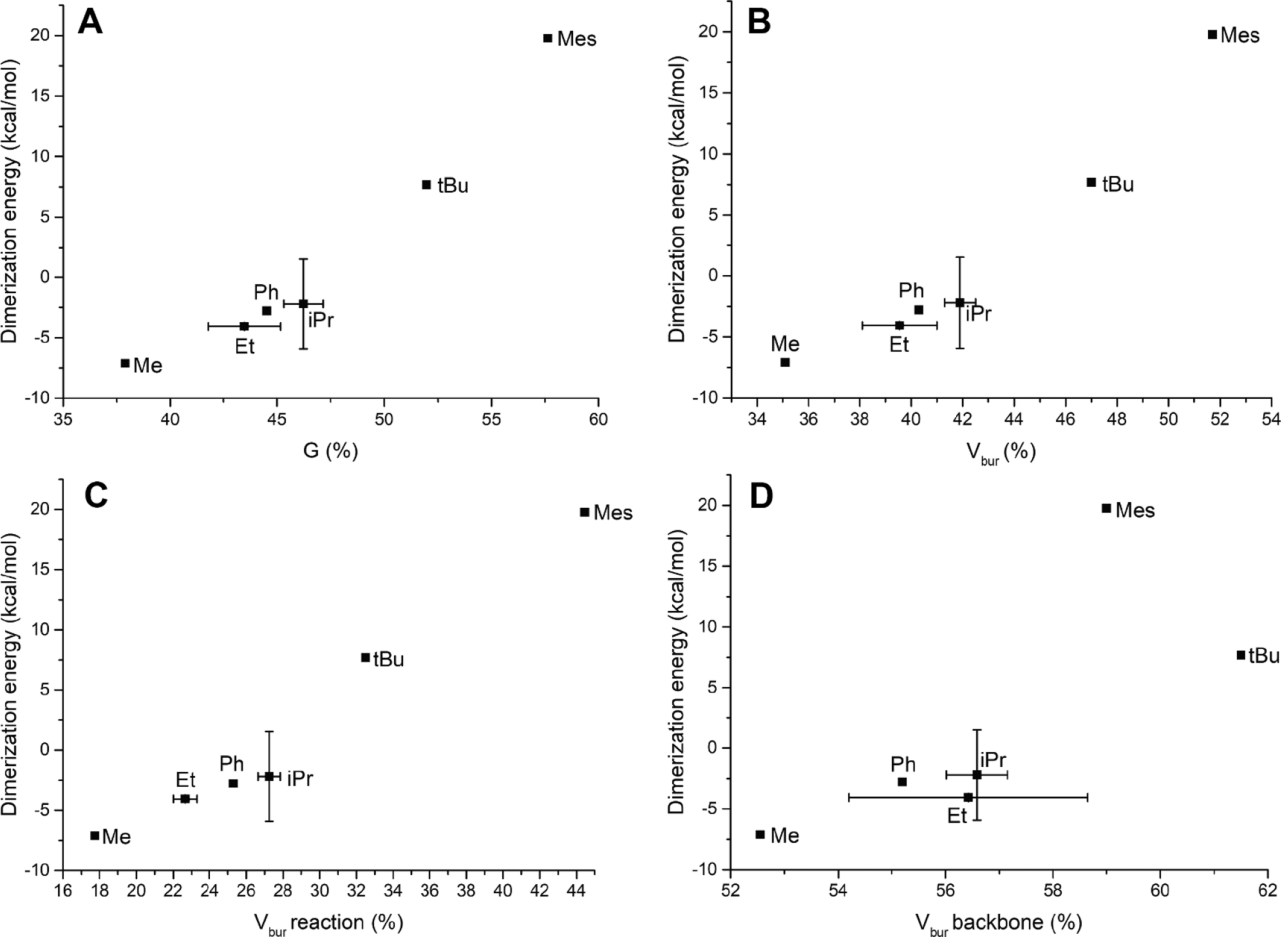
Correlation of the dimerization energy of ^R^(PNNP)*Cu_2_H and different steric parameters. (A) *G*-parameter. (B) Buried volume. (C) Reaction buried volume. (D) Backbone buried volume.

The dimerization energies for ^R^(PNNP*)Cu_2_H, R = Me, Ph, iPr, tBu, and Mes were calculated using DFT and the results plotted against the buried volume and *G-*parameter (Figure 11). These results show the expected trend in which an increase in steric encumbrance also leads to an increase in dimerization energy. In addition, applying the hemisphere analysis shows that there is no clear correlation between the backbone buried volume and the dimerization energy (Figure 11D). In contrast, for the reaction buried volume, a similar correlation as with the total buried volume and *G-*parameter is observed (Figure 11C). This shows that the reaction buried volume is the main contributor to the trend observed in Figure 11 A and B as expected.

In the dimerization equilibria, the [^R^(PNNP*)Cu_2_H]_2_ complexes with iPr and Et substituents were considered as a special cases because the isopropyl and ethyl groups have different steric properties depending on their orientation. In general, the rotational barrier around the C–P bond is low, leading to effectively free rotation at room temperature. Computationally, this is difficult to probe because DFT calculations typically find the local minima that correspond to a specific orientation. To probe how large the influence of such different orientations is, we calculated the dimerization equilibrium (Scheme 3) for three different orientations of the isopropyl groups and two of the ethyl groups (Figures S15 and S16). It should be noted that this does not exhaustively probe the full range of possible dimerization energies and buried volumes caused by the different iPr and Et orientations.^51^ In Figure 11, the average dimerization energies and buried volumes of these conformations are plotted with the error bars to indicate the spread that was found. These results re-iterate the importance of checking how representative the static configuration of a molecule is, before drawing conclusions about the steric bulk using these quantifications. Nevertheless, when the appropriate precautions are taken, the buried volume and *G-*parameter approaches for dinuclear complexes yield useful results for gaining insight into the steric encumbrance of dinuclear complexes.

## Conclusions

In conclusion, we explored a systematic approach for the quantification of the steric parameters of 1,8-napthyridine-based dinucleating ligands. We adapted the buried volume and *G-*parameter approaches for the analysis of 1,8-naphthyridine ligands and investigated the appropriate parameters for the expansion of these methods for their use on dinuclear complexes. The validity of the resulting methods was verified by comparing them to the analogous mononuclear approaches and to the dimerization energies of ^R^(PNNP*)Cu_2_H complexes. This showed that, using the expanded *V*_bur_ and *G-*parameter approaches, the sterics of 1,8-napthyridine-based dinuclear complexes can be reliably calculated. In addition, it was shown that the orientation-dependent analysis of the dinuclear binding pocket is feasible using the hemisphere analysis of *V*_bur_. Readily available software^16,19^ can be applied for calculating these steric parameters, and a pictorial guide for performing these calculations is supplied as Supporting Information.

Applying this approach, we showed that exchanging the phosphine substituents on PNNP expanded pincer ligands provides access to a broad range of steric characteristics for the corresponding complexes. Surprisingly, it was found that the protonation state of the PNNP backbone does not substantially influence the sterics. In contrast, the modification of the linkers between the phosphines and the naphthyridine core, or the metal–metal distance can be used to influence the steric encumbrance of the bimetallic core. Modifications of the ligand backbone do impact the rigidity of the complexes, which in turn affects the flexibility in the corresponding complexes to adopt geometries that feature lower steric encumbrance of the dinuclear core. We envision that this methodology can provide analogous insights into the effect of ligand modifications on other dinuclear complexes, thereby providing a tool to rationally modify chemical reactivity of these complexes through the ligand design.

### Computational Methods

Calculations were performed using ORCA software versions 4.0.1.2 and 4.2.1 (see the Supporting Information for details).^52^

## Supporting Information Available

Computational methods; pictographic guide for performing sterics calculations (*V*_bur_ and *G*); additional data; and numerical data corresponding to the figures in the text (PDF)
Cartesian coordinates of all calculated structures (XYZ)

## Supplementary Material

om2c00458_si_001.pdf

om2c00458_si_002.xyz

## Notes

The output files of the calculations described in this work are openly available as datapackage at https://doi.org/10.4121/20934589. Videographic tutorials on the calculation of the buried volume and the G parameter can be found on https://youtu.be/RQz2vOKM8gE, https://youtu.be/P_jMaLtt-F8 and https://youtu.be/k0OSPIgciBY, respectively.
