# Supplementary material for: Quantification of the Steric Properties of 1,8-Naphthyridine-Based Ligands in Dinuclear Complexes
Source: Organometallics. 2022 Dec 2;42(1):27–37. doi: 10.1021/acs.organomet.2c00458 (PMC9832537; doi:10.1021/acs.organomet.2c00458)
Supplement: Supplementary file 1 — om2c00458_si_001.pdf [file om2c00458_si_001.pdf]

Supporting information “Quantification of the Steric Properties of 1,8-Naphthyridine Based Ligands in Dinuclear Complexes”

*Lars Killian[#], Roel L. M. Bienenmann[#] & Daniël L. J. Broere**

Organic Chemistry and Catalysis, Institute for Sustainable and Circular Chemistry, Faculty of Science, Utrecht University, Universiteitsweg 99, 3584 CG, Utrecht, The Netherlands. Email: d.l.j.broere@uu.nl. [#]Both authors contributed equally to this work.

Contents

General considerations 3
Overlay ^{tBu}(PNNP)Cu_2Cl_2 with reported crystal structure 3
Buried volume calculations using the SambVca 2.1 A Web Application 3
Solid angle calculations using *Solid-G* 6
Expansion of V_{bur} and G parameter to dinuclear complexes 8
Effect of substituents on the steric parameters of PNNP complexes 9
Influence of the Cu–Cu and P–P distance on the steric parameters 10
Dependence of the first coordination sphere size on metal-metal distance 11
Different symmetries in PNNP complexes 13
Methylated ligand 14
Different types of naphthyridine ligands 16
Hydride dimerization equilibrium 17
References: 19

General considerations

Calculations were performed using ORCA software versions 4.0.1.2 (geometries of ^{R}(PNNP)Cu_2Cl_2 where R=Me, Ph, iPr, Cy and tBu) and 4.2.1.[1–3] The Becke 1988 exchange functional[4] was used in combination with the Perdew 1986 correlation functional (BP86)[5]. The redefinition of Ahlrichs triple-zeta split valence basis set (def2-TZVP) was used on all atoms.[6] All calculations except those regarding the hydride dimerization were performed with Grimme's DFT-3 dispersion correction with Becke-Johnson damping.[7,8] Buried volume calculations were performed using the SambVca 2.1A web application following the methodology described below.[9] *G*-parameter calculations were performed using the *Solid-G* software using the methodology detailed below.[10] The starting geometries were obtained by modification of the closest available reported crystal structure.[11–14]

Overlay ^{tBu}(PNNP)Cu_2Cl_2 with reported crystal structure

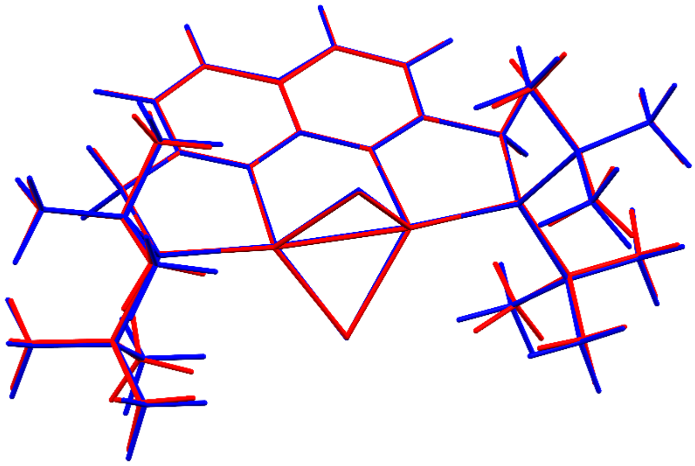

Figure S1: Overlay of the reported[12] crystallographically determined structure of ^{tBu}(PNNP)Cu_2Cl_2 (red) with the calculated structure (blue) showing a good match.

Buried volume calculations using the SambVca 2.1 A Web Application

For calculation of the total buried volume, the XYZ input file can be used directly as obtained from DFT calculations or single-crystal XRD analysis. Calculations requiring reproducible hemisphere analysis or orientation of the steric maps required manipulation of the input file, which was done using Chemcraft software[15] but this can be done with other software as well. To the structure of the molecule was added a dummy atom of arbitrary nature, and the angle between the dummy atom and both metal centers was set to 0 °. Next, the distance between the dummy atom and either metal atom was set to half the metal-metal distance. This structure was saved and used as input file for *SambVca* 2.1. In the *SambVca* 2.1 web application[9], either the dummy atom, or both metal centers can be selected under the option "Select the atoms coordinated to the center of the sphere" and should give identical results. At "Select the atoms for z axis definition", the option "ADD DUMMY" was chosen, and subsequently the previously placed dummy atom, one of the metal centers and the carbon atom of the naphthyridine backbone closest to the midpoint (in between the nitrogen atoms) were selected. This adds a second dummy atom at 90 ° angles to the midpoint dummy atom (the first one to be selected) and the plane made up by the three selected atoms. For the option "Select the atoms for xz-plane definition" either of the metal centers can be selected. Under the option "Select the atoms to be deleted" both dummy atoms, all metal centers and co-ligands are selected and removed (co-ligands can also be removed beforehand, for example in Chemcraft). The sphere radius is the only other parameter that was changed in the *SambVca*

interface. To calculate the buried volume using the ‘metal centric’ approach, one of the metal centers was selected under the option “Select the atoms coordinated to the center of the sphere”.

Step by step guide:

1. For the center of the sphere, select either both copper centers, or the dummy atom to be the center of the sphere.
2. In the next step, click ‘add dummy’ and select the midpoint dummy atom, one of the metal centers and the sp^2 carbon in between both nitrogen atoms (in that order). This will trigger the placement of a new (pink) dummy atom at 90° angles both to the first selected atom (the original dummy atom) and the plane defined by the three selected atoms.
3. For the definition of the xz-plane, select either of the metal centers.
4. Select all dummy atoms, co-ligands and metal centers and click ‘delete selected atoms’.
5. The other options can be ignored, except for the sphere radius, which can be set according to preference (in this case, 5 Å).
6. After submitting the calculation, the steric parameters and steric map are provided in the proper orientation for hemisphere analysis. In this case, the reaction hemisphere is defined by the SW and SE quadrants, and the backbone hemisphere by the NW and NE quadrants. For other orientations, other axes can be defined in step 2 and 3.

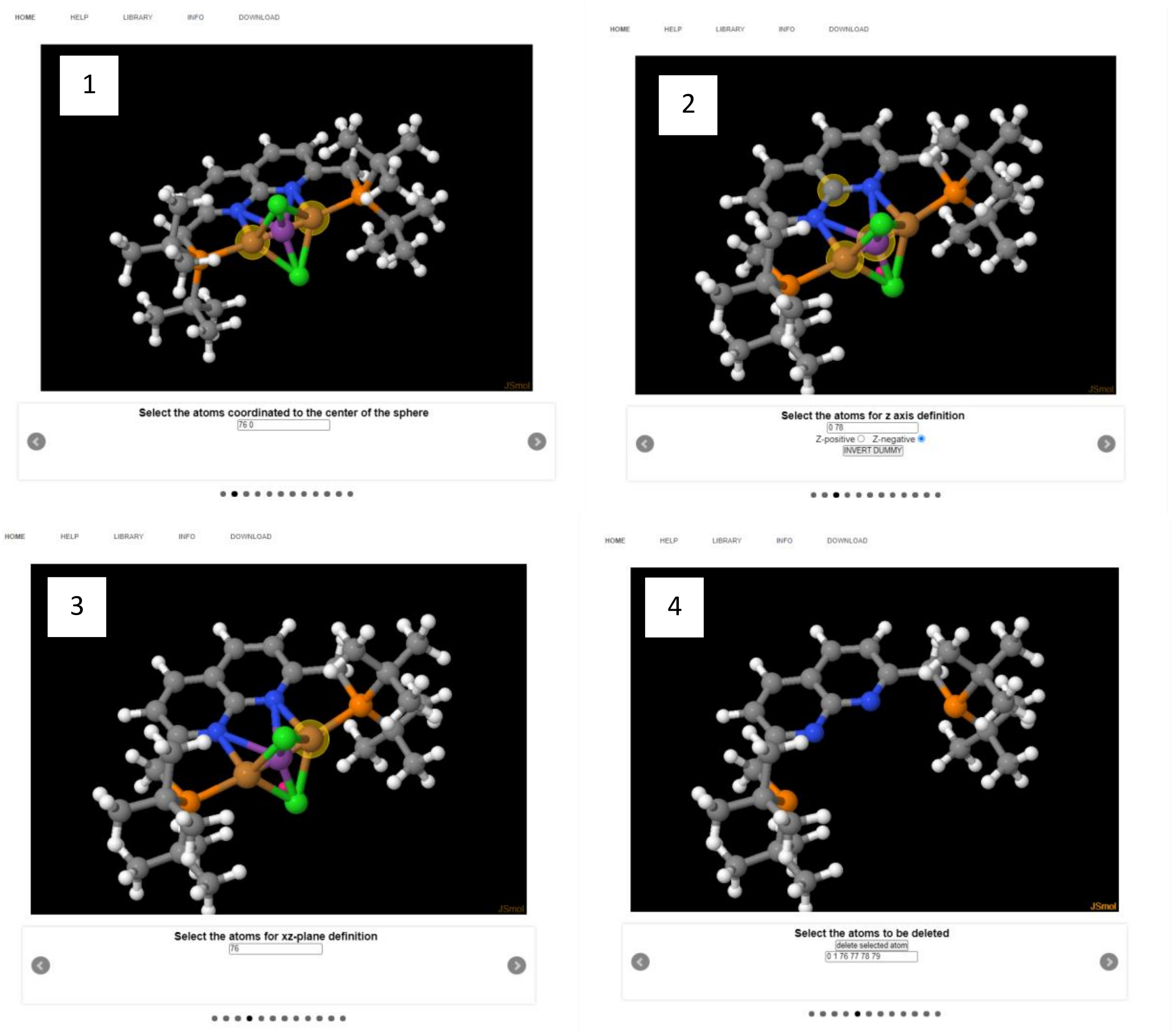

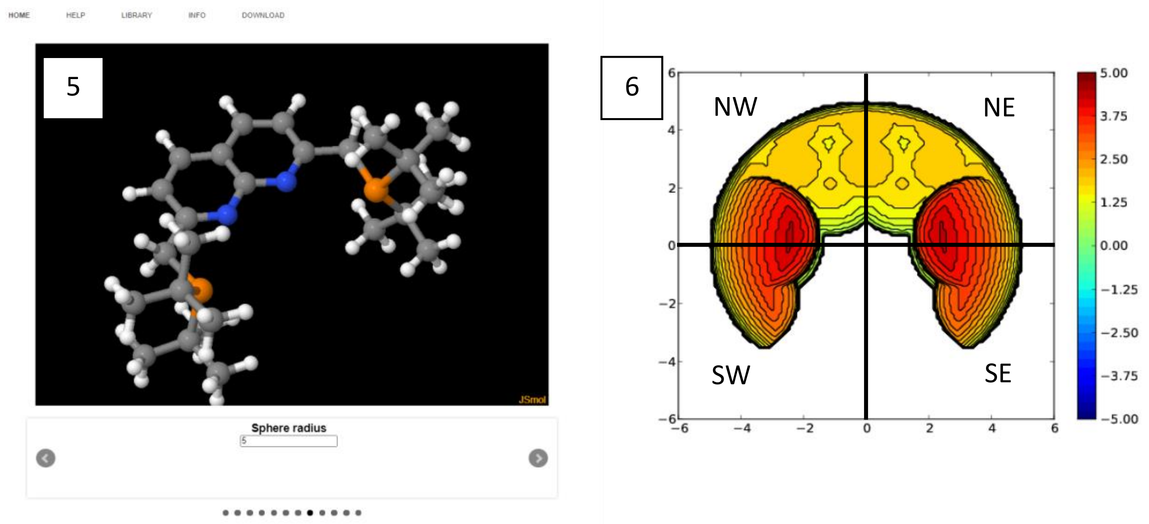

Figure S2: Screenshots accompanying the step by step pictorial guide for calculating the buried volume of dinuclear complexes using the SambVca 2.1 application[9]. Steps: 1: Selecting the central atom, 2: Adding a dummy for z-axis definition, 3: Defining the xy-plane, 4: Deleting redundant atoms, 5: Choosing a sphere radius, and 6: Sterics map output.

Solid angle calculations using *Solid-G*

For the solid angle/*G*-parameter calculations, again a dummy atom was added at the midpoint between both metal centers. The other metal centers and co-ligands were removed beforehand (not strictly necessary). For the metal centered calculations, input files with only one of the metal centers present were made.

The *Solid-G* program[10] requires a specific input file with all atoms numbered uniquely. This requires manipulation of the XYZ file beforehand, which can be done manually or more easily by numbering each atom uniquely in Microsoft Excel. In *Solid-G* the appropriate center of the sphere was chosen in the first step, and the other steps were done with standard settings.

Step by step guide:

1. Open the file with the XYZ coordinates in the *Solid-G* program. Make sure the atoms are labelled individually (C1, C2, C3 etc.). If the program you are using to generate the XYZ file does not have this as a standard option, use a text editor to change the XYZ file. Additionally, the file with the dummy atom as explained before is used, where the original metal centers and co-ligands are removed.
2. Type the label of the dummy atom “Bi1” and click ‘Select Atom’ to make the atom the center of the sphere.
3. Click ‘Identify Ligands’. Check if all the atoms belonging to the ligand are labelled with the same number in the column ‘Ligand’ and if any of these atoms say ‘yes’ in the column ‘Bound?’.
4. Click ‘Calculate Angles’. This loads the result page.
5. To see the shading of the ligand on the sphere, click ‘Start Viewer’.

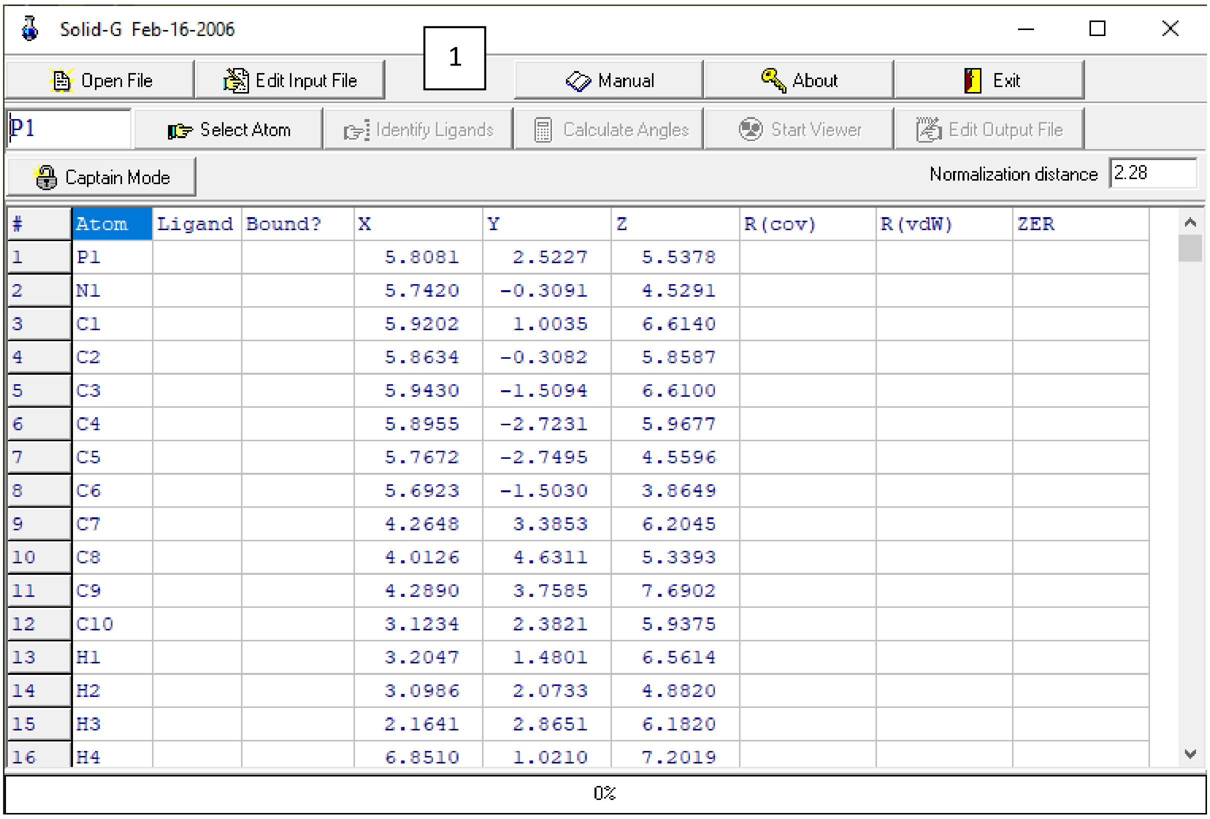

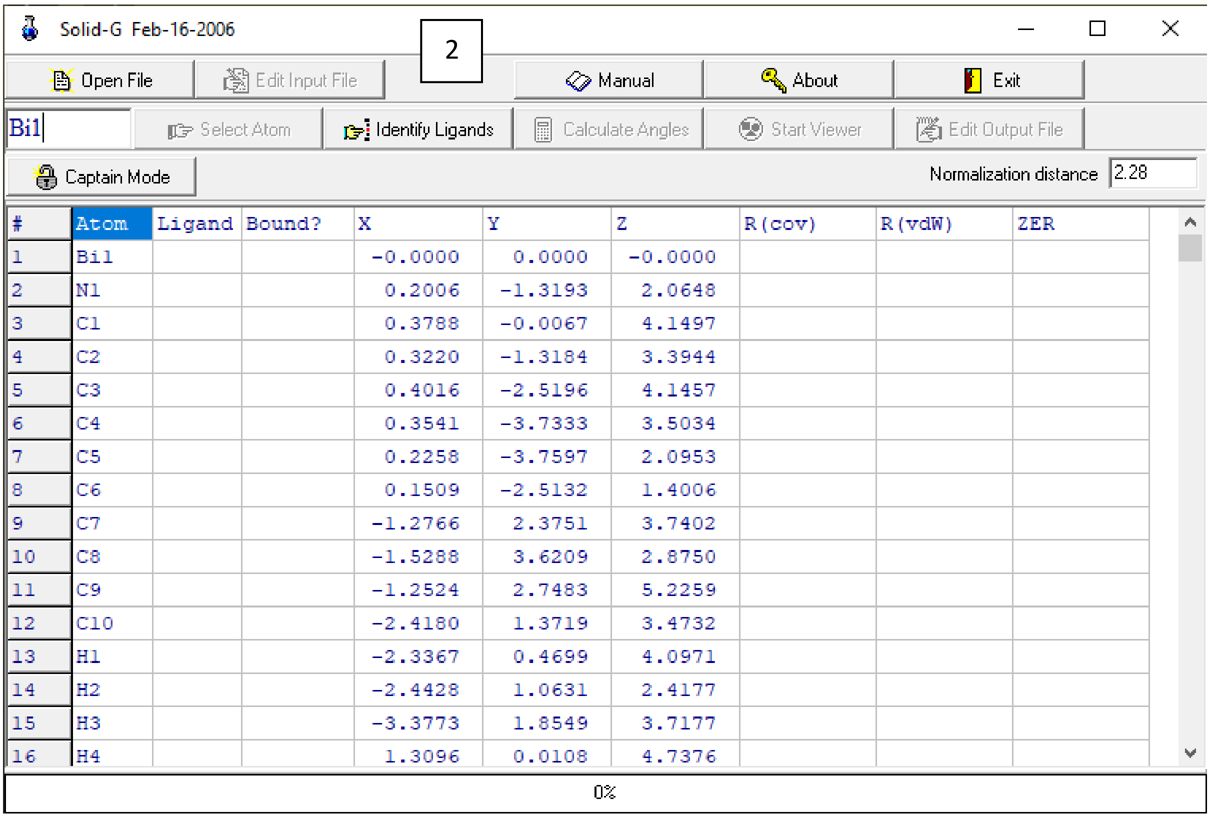

#	Atom	Ligand	Bound?	X	Y	Z	R(cov)	R(vdW)	ZER
1	Bi1			-0.0000	0.0000	-0.0000			
2	N1			0.2006	-1.3193	2.0648			
3	C1			0.3788	-0.0067	4.1497			
4	C2			0.3220	-1.3184	3.3944			
5	C3			0.4016	-2.5196	4.1457			
6	C4			0.3541	-3.7333	3.5034			
7	C5			0.2258	-3.7597	2.0953			
8	C6			0.1509	-2.5132	1.4006			
9	C7			-1.2766	2.3751	3.7402			
10	C8			-1.5288	3.6209	2.8750			
11	C9			-1.2524	2.7483	5.2259			
12	C10			-2.4180	1.3719	3.4732			
13	H1			-2.3367	0.4699	4.0971			
14	H2			-2.4428	1.0631	2.4177			
15	H3			-3.3773	1.8549	3.7177			
16	H4			1.3096	0.0108	4.7376			

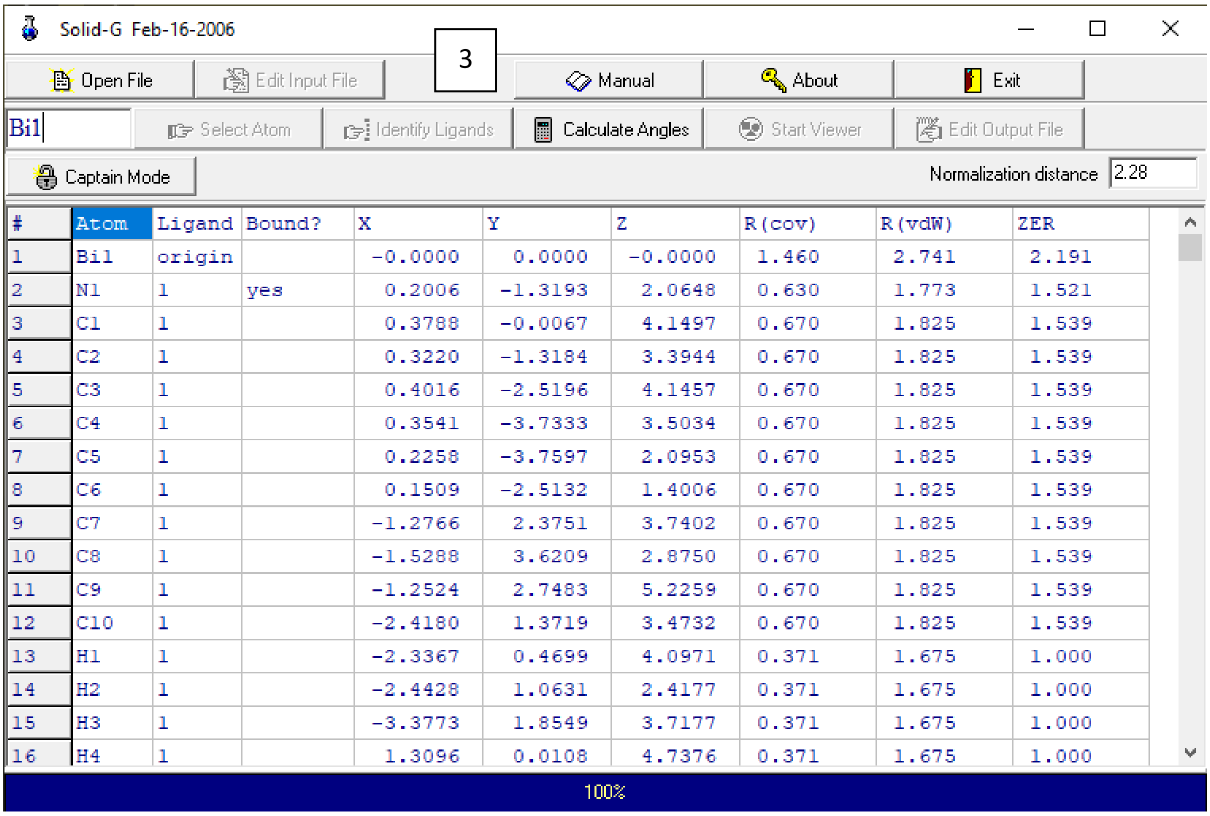

#	Atom	Ligand	Bound?	X	Y	Z	R(cov)	R(vdW)	ZER
1	Bi1	origin		-0.0000	0.0000	-0.0000	1.460	2.741	2.191
2	N1	1	yes	0.2006	-1.3193	2.0648	0.630	1.773	1.521
3	C1	1		0.3788	-0.0067	4.1497	0.670	1.825	1.539
4	C2	1		0.3220	-1.3184	3.3944	0.670	1.825	1.539
5	C3	1		0.4016	-2.5196	4.1457	0.670	1.825	1.539
6	C4	1		0.3541	-3.7333	3.5034	0.670	1.825	1.539
7	C5	1		0.2258	-3.7597	2.0953	0.670	1.825	1.539
8	C6	1		0.1509	-2.5132	1.4006	0.670	1.825	1.539
9	C7	1		-1.2766	2.3751	3.7402	0.670	1.825	1.539
10	C8	1		-1.5288	3.6209	2.8750	0.670	1.825	1.539
11	C9	1		-1.2524	2.7483	5.2259	0.670	1.825	1.539
12	C10	1		-2.4180	1.3719	3.4732	0.670	1.825	1.539
13	H1	1		-2.3367	0.4699	4.0971	0.371	1.675	1.000
14	H2	1		-2.4428	1.0631	2.4177	0.371	1.675	1.000
15	H3	1		-3.3773	1.8549	3.7177	0.371	1.675	1.000
16	H4	1		1.3096	0.0108	4.7376	0.371	1.675	1.000

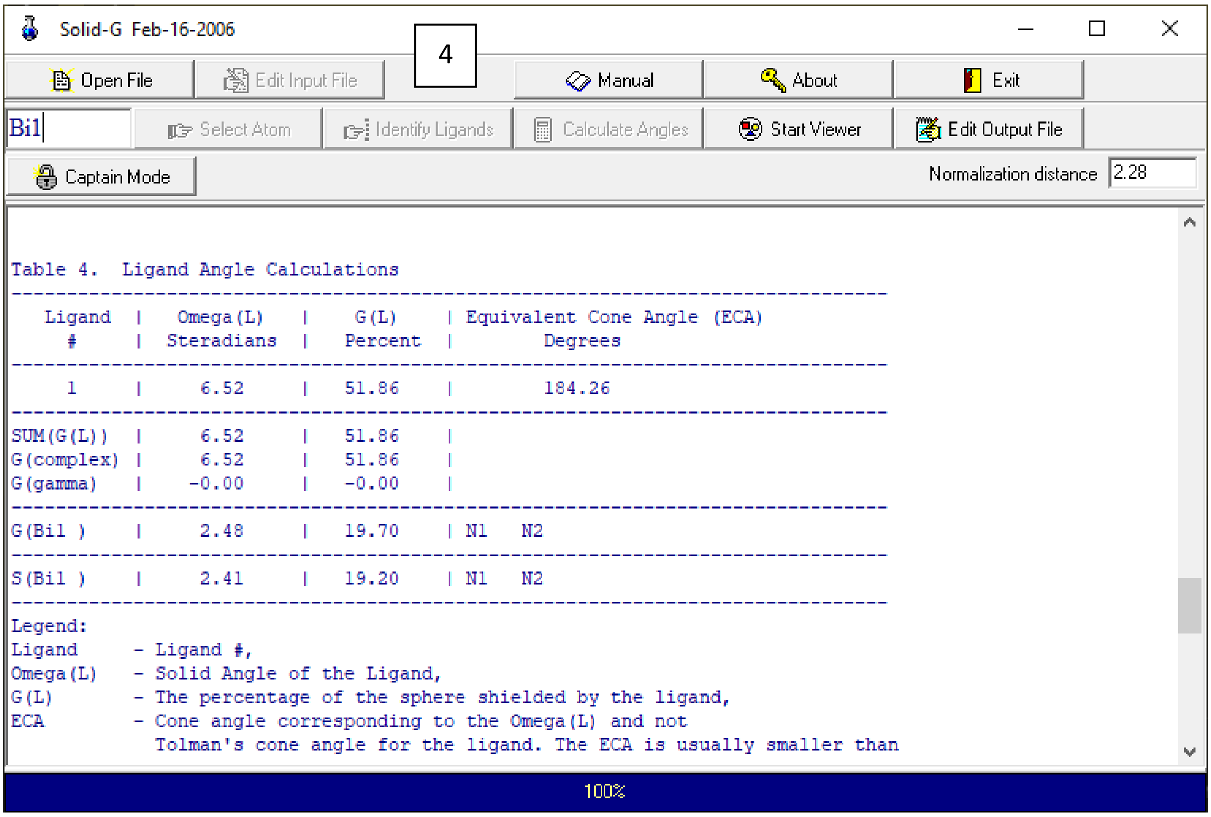

Figure S3: Screenshots accompanying the step by step pictorial guide for calculating the G-parameter of dinuclear complexes using the Solid-G application[10]. Steps: 1: Loading xyz-coordinates, 2: Selecting the central atom, 3: Identifying the ligands, 4: Analyzing the output and G(%) value.

Expansion of V_{bur} and G parameter to dinuclear complexes

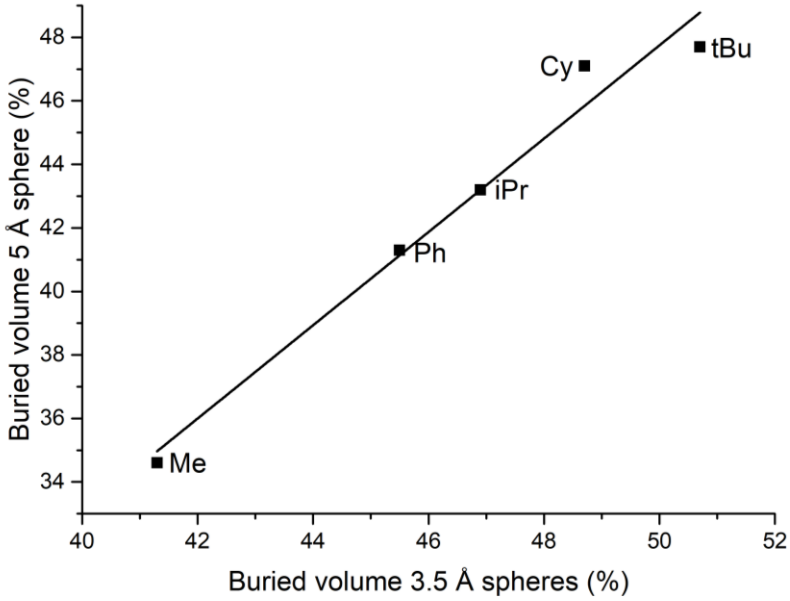

Figure S4: The correlation (R^2=0.965) between the buried volume for different ^{R}(PNNP)Cu_2Cl_2 complexes calculated with a 5Å sphere between the two metal centers versus the buried volume of the same complexes calculated with a 3.5Å sphere on one of the metal centers.

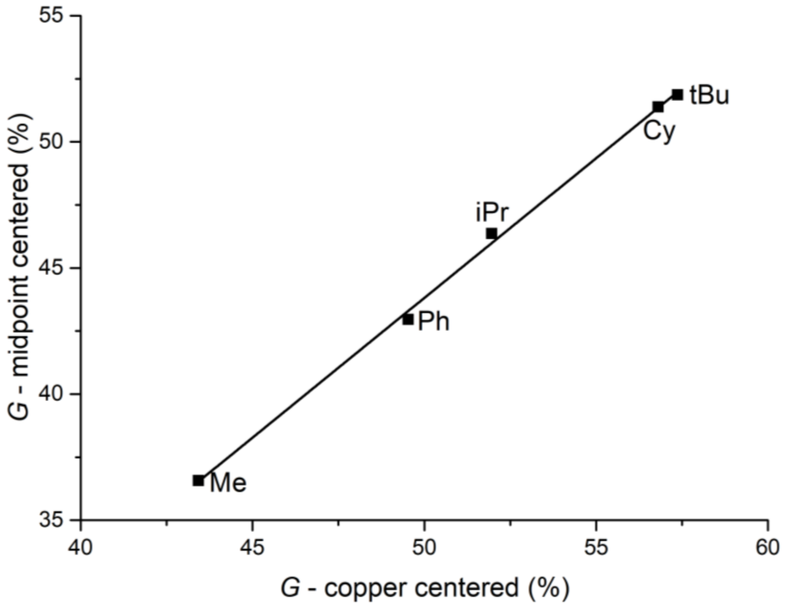

Figure S5: The correlation (R^2=0.998) between the G-parameter for different ^{R}(PNNP)Cu_2Cl_2 complexes calculated with the sphere origin between the two metal centers versus the same complexes calculated with the sphere origin on one of the metal centers.

Effect of substituents on the steric parameters of PNNP complexes

Table S1: Steric parameter for DFT optimized geometries of ^{R}(PNNP)Cu_2Cl_2 complexes, the data for figure 6 in the main text.

**Compound**	**V_{bur} (%)**	**V_{bur} reaction (%)**	**V_{bur} backbone (%)**	**G (%)**	**Twist/tilt**
**^{H}(PNNP)Cu_2Cl_2**	30.4	11.2	49.7	32.6	C_2/No
**^{Me}(PNNP)Cu_2Cl_2**	34.6	18.4	50.8	36.6	No/No
**^{Ph}(PNNP)Cu_2Cl_2**	41.3	24.0	58.6	43.0	No/Yes
**^{iPr}(PNNP)Cu_2Cl_2**	43.2	25.7	60.8	46.4	No/Yes
**^{oTol}(PNNP)Cu_2Cl_2**	46.4	28.8	64.2	49.0	Asymmetric/No
**^{C6F5}(PNNP)Cu_2Cl_2**	46.9	34.8	59.0	51.8	C_s/Yes
**^{Cy}(PNNP)Cu_2Cl_2**	47.1	28.0	66.2	51.4	Asymmetric /No
**^{tBu}(PNNP)Cu_2Cl_2**	47.7	33.4	62.1	51.9	No/No
**^{Mes}(PNNP)Cu_2Cl_2**	50.3	39.0	61.8	55.7	C_s/Yes

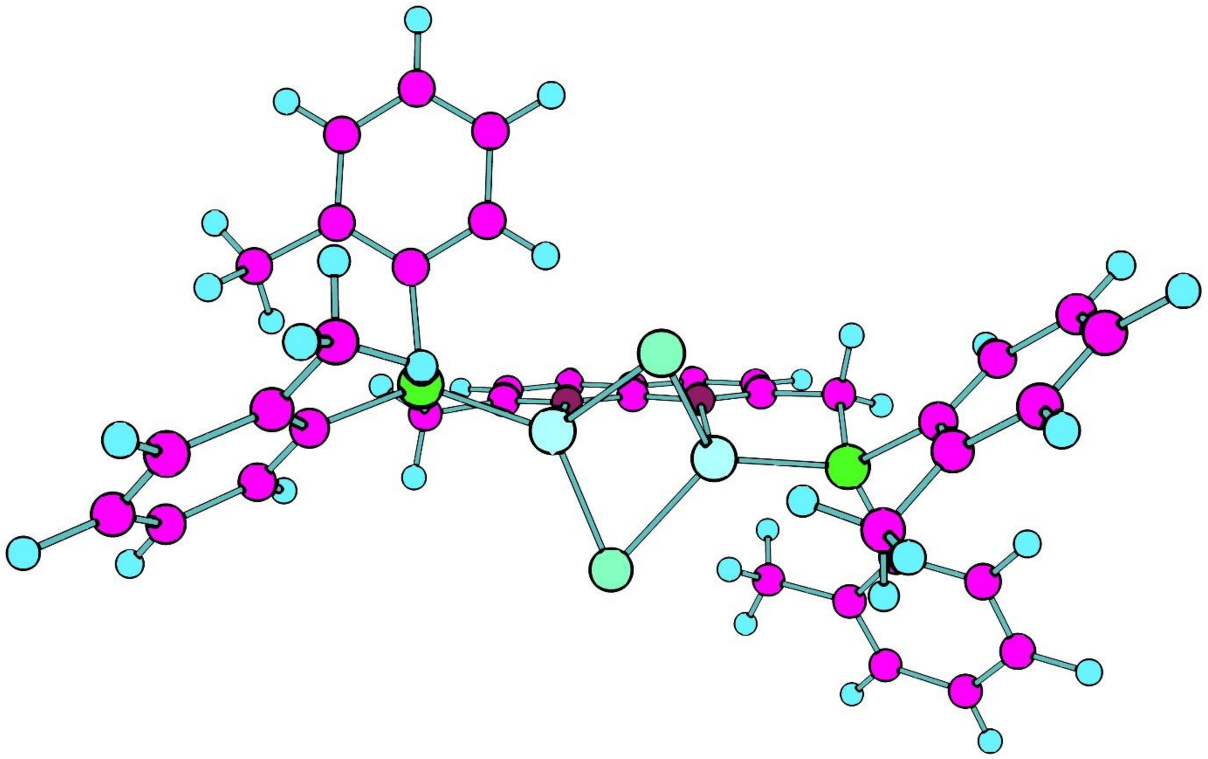

Figure S6: The optimized geometry of ^{oTol}(PNNP)Cu_2Cl_2, showing that two of the methyl groups on the oTol substituents are pointing to the back of the complex (top left and bottom right), and two are pointing toward the center of the complex.

Influence of the Cu–Cu and P–P distance on the steric parameters

Table S2: Steric parameters for different ^{R}(PNNP)Cu_2Cl_2 and ^{R}PONNOPCu_2Cl_2 complexes. Full data for table 1 in the main text.

**Compound**	**V_{bur} (%)**	**V_{bur} rxn (%)**	**V_{bur} backbone (%)**	**G (%)**	**P – P distance (Å)**	**Cu – Cu distance (Å)**
**^{iPr}(PNNP)Cu_2Cl_2**	43.2	24.8	60.8	46.4	6.87	2.55
**^{iPr}(PONNOP)Cu_2Cl_2**	41.8	22.3	61.3	45.92	6.84	2.60
**^{tBu}(PNNP)Cu_2Cl_2**	47.7	33.4	62.1	51.9	6.86	2.53
**^{tBu}(PONNOP)Cu_2Cl_2**	45.5	27.6	63.4	49.8	6.85	2.59
**^{Ph}(PNNP)Cu_2Cl_2**	41.3	24.0	58.6	43.0	6.87	2.57
**^{Ph}(PONNOP)Cu_2Cl_2**	39.5	24.1	54.9	42.1	6.83	2.59

Table S3: Steric parameter for ^{tBu}(PNNP) complexes in various protonation states. Full data for table 2 and figure 7 in the main text.

**Compound**	**V_{bur} (%)**	**V_{bur} rxn (%)**	**V_{bur} backbone (%)**	**G (%)**	**P – P distance (Å)**	**Cu – Cu distance (Å)**
**^{tBu}(PNNP)Cu_2O^tBu**	45.7	28.9	62.7	49.8	6.89	2.81
**^{tBu}(PNNP)*Cu_2O^tBu**[a,b]	42.7	25.0	60.5	46.7	7.29	3.03
**^{tBu}(PNNP)*Cu_2O^tBu**	45.6	29.4	62.1	50.6	6.94	2.77
**^{tBu}(PNNP)**Cu_2O^tBu**[a]	43.5	27.2	59.9	47.2	7.25	2.96

**^{tBu}(PNNP)**Cu_2O^tBu**	44.5	28.6	60.3	50.3	7.14	2.59
**^{tBu}(PNNP)Cu_2Mes**	48.5	34.7	62.2	52.7	6.78	2.38
**^{tBu}(PNNP)*Cu_2Mes**	48.7	35.2	62.2	53.5	6.81	2.36
**^{tBu}(PNNP)**Cu_2Mes**	48.9	36.0	61.9	54.2	6.81	2.34

[a]Reported crystal structure was used.[12] [b]The average value for both molecules in the asymmetric unit cell was taken.

Influence of M-M distance on other ligands

In order to assess the influence of the M-M distance on the steric parameters for other 1,8-naphthyridine ligands, we investigated the steric properties of three cobalt complexes reported by Uyeda and co-workers.[16] The reported solid state structures were used for the calculations of the buried volume and *G*-value (table S4). An important difference between the NDI and the PNNP system is the flexibility. In the PNNP system, a change in M-M distance also caused a similar change in P-P distance. In the NDI system, however, the ligand twists upon increasing M-M distance and the N-N distance hardly changes. The M-M distance in this case therefore also does not correlate with the steric parameters in the way it did for the PNNP system.

Table S4: Steric parameters for different dicobalt NDI complexes as reported by Uyeda and co-workers[16], calculated using the reported structures determined by XRD.

Compound	V_{bur} (%)	V_{bur} rxn (%)	V_{bur} backbone (%)	G (%)	N – N distance (Å)	M – M distance (Å)
**(NDI)Co_2Cl_3(THF)**	45.8	35.4	56.15	51.0	6.58	3.54
**(NDI)$Co_2Cl(C_6D_6)$**	42.5	30.7	54.5	51.0	6.21	2.41
**(NDI)Co_2Cl(alllyl)**	42.1	29.3	55.0	50.6	6.36	2.62

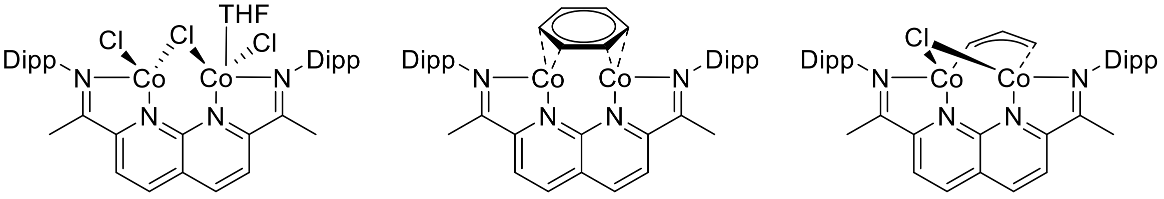

(NDI)Co_2Cl_3(THF) (NDI)$Co_2Cl(C_6D_6)$ (NDI)Co_2Cl(alllyl)

Figure S7: Structures of various dicobalt NDI complexes as reported by Uyeda and co-workers. [16]

Dependence of the first coordination sphere size on metal-metal distance

In the buried volume calculations, the sphere with a radius of 5 Å centered at the midpoint between the two metal centers serves as a reasonable first coordination sphere. Given the large dependence of the steric environment on metal-metal distance, we reasoned that, changing this sphere size depending on the metal-metal distance seemed intuitive.

When again looking at Figure 3, the V_{bur} (%) does not have a large dependence on the sphere radius, as long as this radius is between 3.5 and 5.5 Å. Typical metal-metal distances in the expanded pincer ligand are roughly between 2 and 3 Å. When one considers a sphere that encompasses both 3.5 Å spheres on the metal atoms (Figure 2), this difference of 1 Å would lead to a change in sphere radius of 0.5 Å leading to only minimal differences in V_{bur} (%). Sphere radii adjusted in this way were used to calculate the buried volume for three ^{tBu}(PNNP) dicopper complexes with Cu–Cu distances between 2.5 and 3.0 Å (Table S4). Indeed, the adjusted buried volumes are only marginally different

from the original buried volumes. The overall trend in buried volume depending on the sphere radius is also similar for these complexes, as shown in Figure S4. This confirms that the effect of this type of adjustment of the sphere radius is indeed minimal and hence the choice for a 5 Å sphere seems robust.

*Table S5: The effect of changing the sphere size according to the metal-metal distance on the percentual and absolute buried volumes. Adjusted sphere radii are calculated as 3.5 + 0.5 * M – M distance.*

**Compound**	**V_{bur} 5 Å (%)**	**V_{bur} 5 Å (Å^3)**	**Cu – Cu distance (Å)**	**Adjusted Sphere radius (Å)**	**V_{bur} adjusted (%)**	**V_{bur} adjusted (Å^3)**
**^{tBu}(PNNP)Cu_2Cl_2**	47.7	250	2.53	4.8	47.8	221
**^{tBu}(PNNP)*Cu_2O^tBu**[a,b]	42.7	224	3.03	5.0	42.7	224
**^{tBu}(PNNP)**Cu_2Mes**	49.0	256	2.34	4.7	49.1	214

[a]Crystal structure was used.[12] [b]The average value for both molecules in the asymmetric unit cell was taken.

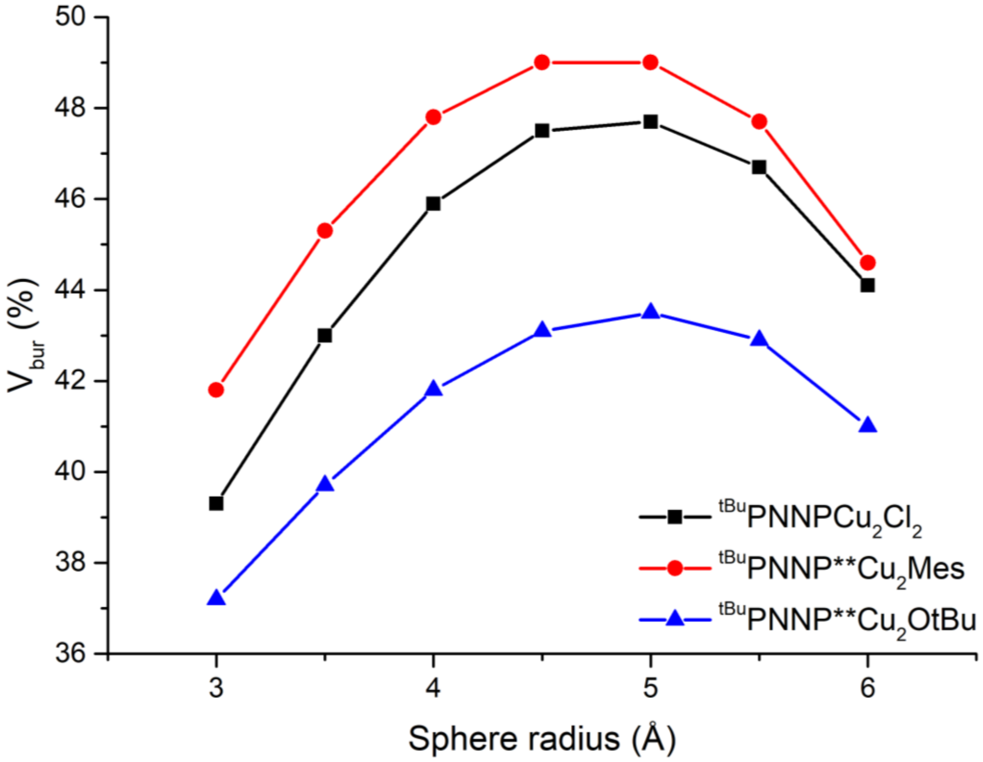

Figure S8: Comparison in sphere radius dependence on the buried volumes for three tBu(PNNP) dicopper complexes with varying metal-metal distances.

Different symmetries in PNNP complexes

To examine to which extend the twists and tilts of PNNP complexes impact the steric encumbrance, the optimized geometry of some of the ^{R}(PNNP)Cu_2Cl_2 complexes with R = Ph, Cy and C_6F_5 was taken (figure S6), and the R groups were changed to *tert*-butyl groups while leaving the rest of the molecular coordinates fixed. The geometry of only the R groups was then optimized while the rest of the geometry was frozen. The results of these calculations (table S5) provide an indication of the effect of these geometrical changes on the steric encumbrance.

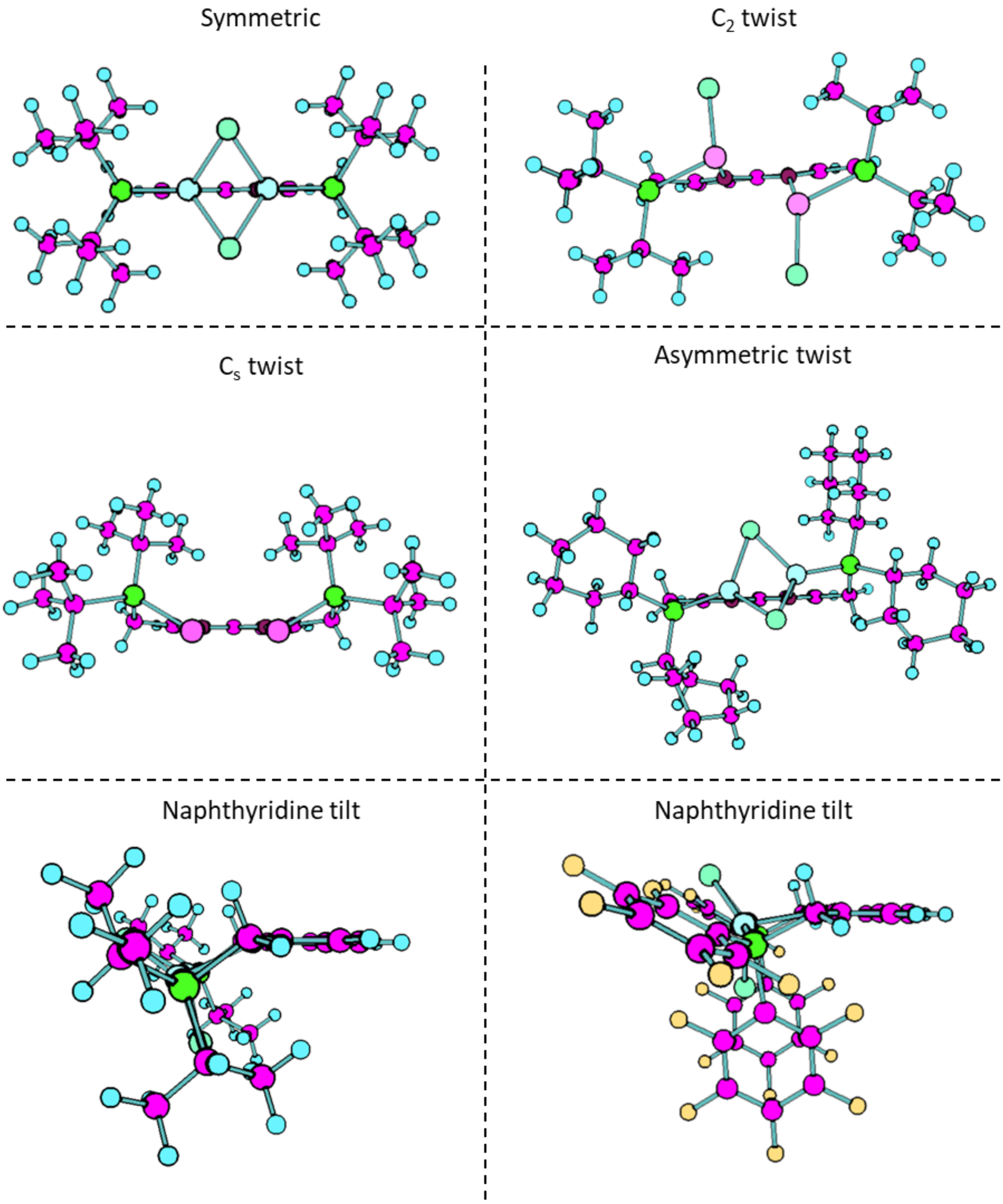

Figure S9: Different twists and tilts observed in expanded pincer ligands, taken from a variety of structures, both computational and crystal structures. Some co-ligands are omitted for clarity.

Table S6: The effect of different conformations on the total buried volumes, and the buried volumes of the reaction and backbone hemispheres.

Compound	V_{bur} (%)	V_{bur} rxn (%)	V_{bur} backbone (%)	G (%)
**^{tBu}(PNNP)Cu_2Cl_2**	47.7	33.4	62.1	51.9
**^{tBu}(PNNP)Cu_2Cl_2 with naphthyridine tilt[a]**	47.0	31.4	62.7	50.8
**^{tBu}(PNNP)Cu_2Cl_2 with asymmetric twist[b]**	47.1	29.3	65.0	50.9
**^{tBu}(PNNP)Cu_2Cl_2 with C_s twist[c]**	47.0	30.3	63.7	51.1

[a]As obtained from the computational structure of ^{Ph}(PNNP)Cu_2Cl_2. [b]As obtained from the computational structure of ^{Cy}(PNNP)Cu_2Cl_2. [c]As obtained from the computational structure of ^{C6F5}(PNNP)Cu_2Cl_2.

Methylated ligand

For the complex with methyl substituted phosphines, the methylated structure features a tilted naphthyridine plane as opposed to the C_{2V} symmetric non-methylated structure (Figure S7). This causes the copper atoms to be below the naphthyridine plane, but above the P–P line, which creates in effect a similar situation as with the other structures. In this case, the copper atoms are pulled toward each other by the bridging chlorides and the naphthyridine N-atoms on one hand, while being pulled apart by the phosphines that are now not in line with the copper atoms due to the induced tilt. This then leads to a net elongation of the Cu–Cu distance with respect to the non-methylated structure. For the H substituted analogue, this effect is not present since the methyl groups are too far from the Hs on the phosphines to effect such a change.

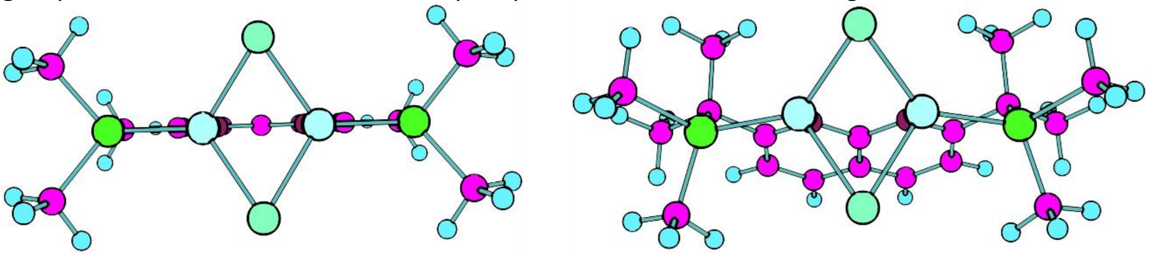

Figure S10: The optimized structure of the non-methylated ^{Me}(PNNP)Cu_2Cl_2 (left) and the methylated version (right).

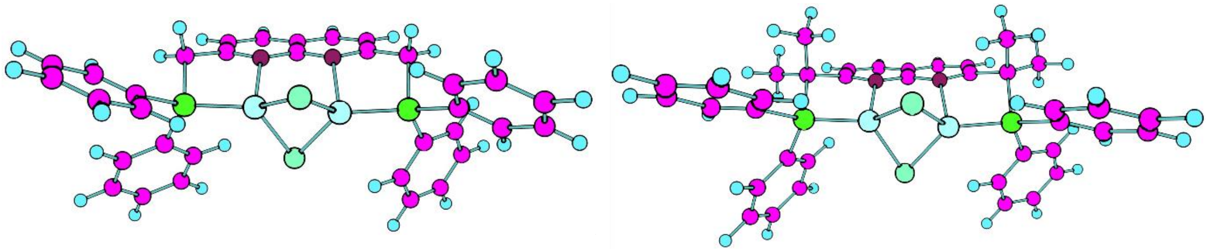

Figure S11: The optimized structure of the non-methylated ^{Ph}(PNNP)Cu_2Cl_2 (left) and the methylated version (right).

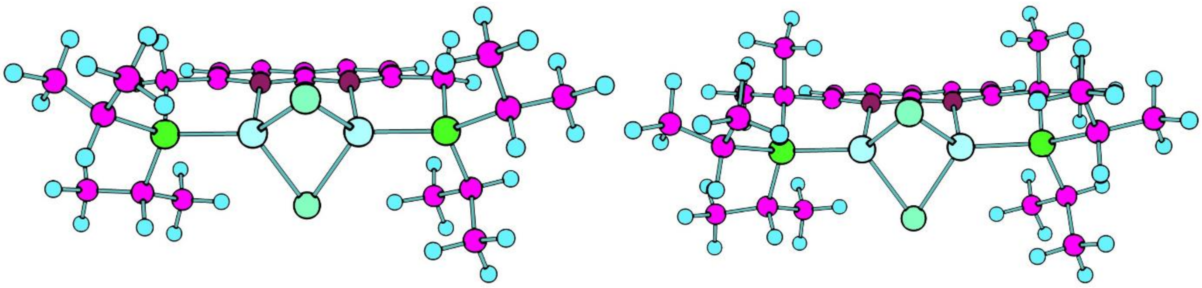

Figure S12: The optimized structure of the non-methylated $^{iPr}(PNNP)Cu_2Cl_2$ (left) and the methylated version (right).

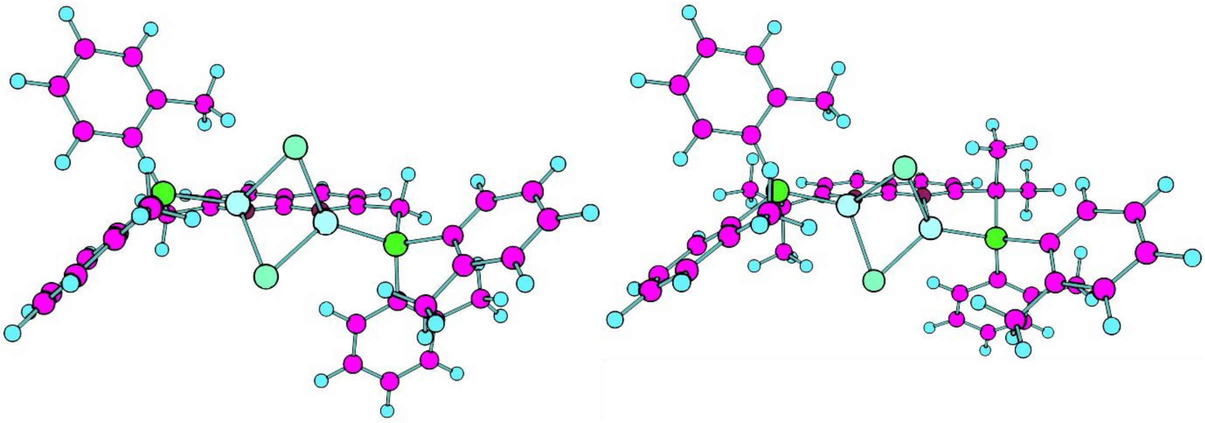

Figure S13: The optimized structure of the non-methylated $^{oTol}(PNNP)Cu_2Cl_2$ (left) and the methylated version (right).

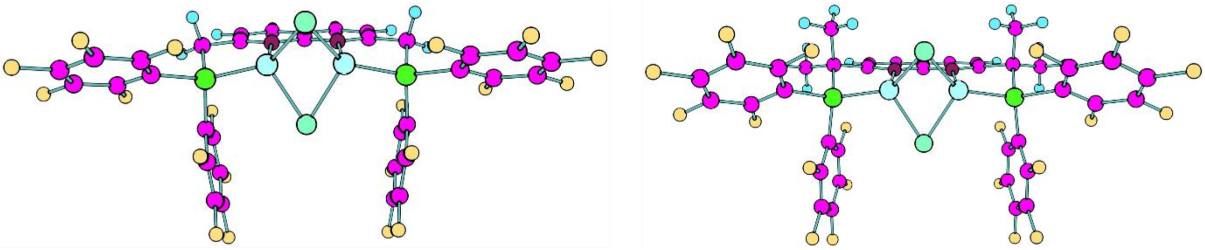

Figure S14: The optimized structure of the non-methylated $^{C6F5}(PNNP)Cu_2Cl_2$ (left) and the methylated version (right).

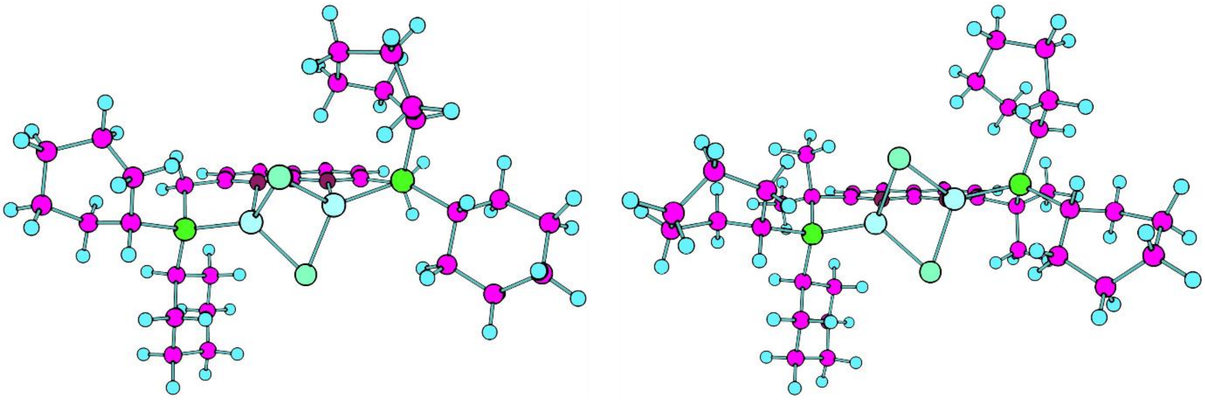

Figure S15: The optimized structure of the non-methylated $^{Cy}(PNNP)Cu_2Cl_2$ (left) and the methylated version (right).

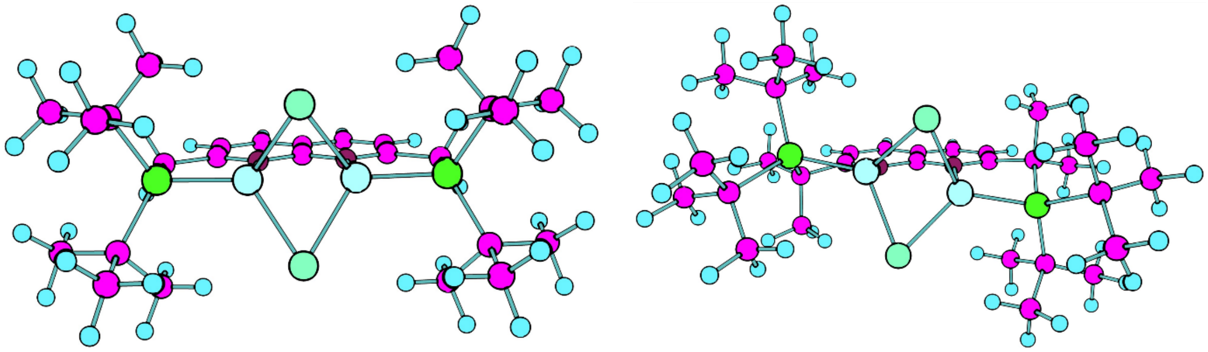

Figure S16: The optimized structure of the non-methylated ^{tBu}(PNNP)Cu_2Cl_2 (left) and the methylated version (right).

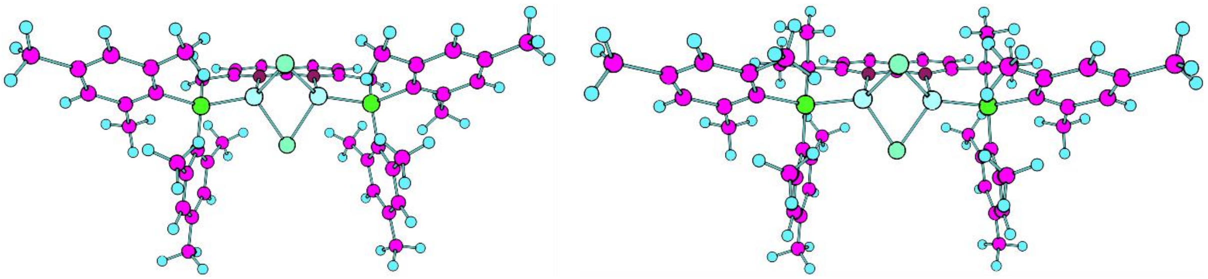

Figure S17: The optimized structure of the non-methylated ^{Mes}(PNNP)Cu_2Cl_2 (left) and the methylated version (right).

Different types of naphthyridine ligands

Table S7: The steric parameters of different types of 1,8-naphthyridine based ligands from literature.

Compound	V_{bur} (%)	V_{bur} rxn (%)	V_{bur} backbone (%)	G (%)
**^{tBu}(PNNP)Cu_2Cl_2**[12]	46.5	31.5	61.4	46.7
**[(DPFN)Cu_2(MeCN)] BPh_4**[17]	49.3	35.3	63.3	51.5
**^{dipp}(NDI)Ni_2(C_6H_6)**[18]	42.0	29.9	54.1	51.1
**(bpnp)Cu_2Cl_2**[19]	29.9	11.7	48.1	32.1

Hydride dimerization equilibrium

For calculating the dimerization energies for ^{R}(PNNP*)Cu_2H (scheme 3 and figure 11) the geometries of the monomer and the dimer were optimized (BP86/def2-TZVP) and the Gibbs free energy of dimerization was calculated. Then the geometry of the monomer was used to calculate the steric parameters (i.e. V_{bur} and *G*, Table S7) as described before.

The dimerization energies are calculated without dispersion correction in the DFT method. Dispersion correction, in the case of the [^{R}(PNNP*)$Cu_2H]_2$ dimers, overestimates the dispersion energy and hence also the dimerization energy. This is evident when comparing the crystal structure of [^{tBu}(PNNP*)$Cu_2H]_2$ with the optimized structure with and without dispersion correction as was reported before.[12] Using a method without dispersion correction does yield a structure that corresponds well to the crystal structure, however, a positive dimerization energy is calculated for the [^{tBu}(PNNP*)$Cu_2H]_2$ complex (i.e. dimerization costs energy). This is contradictory to the experimental observations that show that this complex is a dimer both in solution and in solid state.[12] The dimerization energy that is calculated without dispersion correction is offset by the true dispersive energy (that is by definition attractive), which is why the calculated dimerization energies are higher than they are in reality. Given that the calculated structure without dispersion correction more closely resembles the experimentally determined structure than the one calculated with dispersion correction, we infer that the error introduced by the dispersion correction is larger than the one created by leaving this correction out, also in the case of the dimerization energies. As a potential alternative, we considered using the geometries of calculated structures without dispersion correction, and perform a single point calculation with dispersion correction. This does lead to a negative dimerization energy (-41.8 kcal/mol) for the tBu structure, however, reasoning along the same lines as above, we suspect that the error introduced by the dispersion correction is still quantitatively larger than the one introduced without it.

Additionally, Basisset Superposition Error (BSSE) was investigated as a potential cause for the overestimation of the dimerization energy. To get an estimate for the magnitude of this BSSE, a counterpoise correction was used as described in the Orca 4.2.1 manual (8.1.6)[20] for the ^{tBu}(PNNP)*Cu_2H structures calculated without dispersion correction. This yielded a BSSE of 3.6 kcal/mol, indicating that this error is likely too small to account for the discrepancy we found between the experiment and the calculated value. In addition, we performed a geometry optimization with a geometrical Counterpoise Correction (gCP) as described in the Orca 4.2.1 manual (9.3.2.13)[20] and GD3BJ dispersion correction, and this yielded a similarly distorted structure as was found without the counterpoise correction (Figure S15). From this we conclude that the BSSE is not the main cause for the observed error in the calculated dimerization energy.

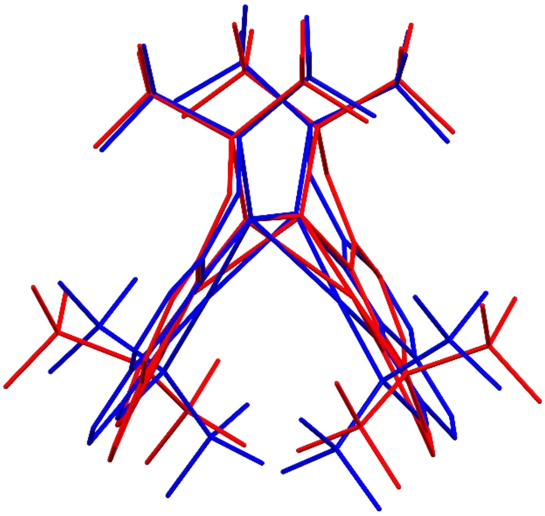

Figure S18: Overlay of the reported[12] crystallographically determined structure of [^{tBu}(PNNP)Cu_2H]$_2$ (red) with the calculated structure with dispersion correction and geometrical counterpoise correction (blue) showing that there is still a mismatch.*

For the isopropyl ^{R}(PNNP*)Cu_2H complexes, three different conformations of the monomer and three of the dimer were calculated. For calculating the sterics parameters, the three monomer structures were used, leading to three values for each parameter. These three were then averaged and this average was plotted. The error bars shown in figure 11 show the largest deviation of one of the single values from the average since it is to indicate an order of magnitude of the error introduced by these different conformations. For calculating the dimerization energies, first the dimerization energy of the three pairs of different conformers was calculated. Then, these were averaged and that average was plotted. To obtain the error bars, the largest deviation from the average of one of the three dimerization energies was taken and this was plotted as the error. The analogous method was applied for the ethyl substituents, but in this case with two different conformations.

Table S8: The buried volume (%) of ^{R}(PNNP)Cu_2H complexes per hemisphere, for the reaction hemisphere and backbone hemisphere, and the total buried volume as well as the G-parameter. For Et and iPr, the numbers between brackets represent different configurations that are not symmetry equivalent (Figure S15 and S16).*

	V_{bur} reaction (%)	V_{bur} backbone (%)	V_{bur} (%)	G (%)
**Me**	17.8	34.1	35.1	37.9
**Et(1)**	23.3	40.1	41.0	45.2
**Et(2)**	22.0	37.9	38.1	41.8
**Ph**	25.3	39.4	40.3	44.5
**iPr(1)**	23.3	38.3	41.6	45.7
**iPr(2)**	27.2	43.2	41.6	45.9
**iPr(3)**	27.9	44.0	42.5	47.2
**tBu**	32.5	47.4	47.0	52.0
**Mes**	44.5	53.2	51.7	57.6

Different conformations of Et and iPr

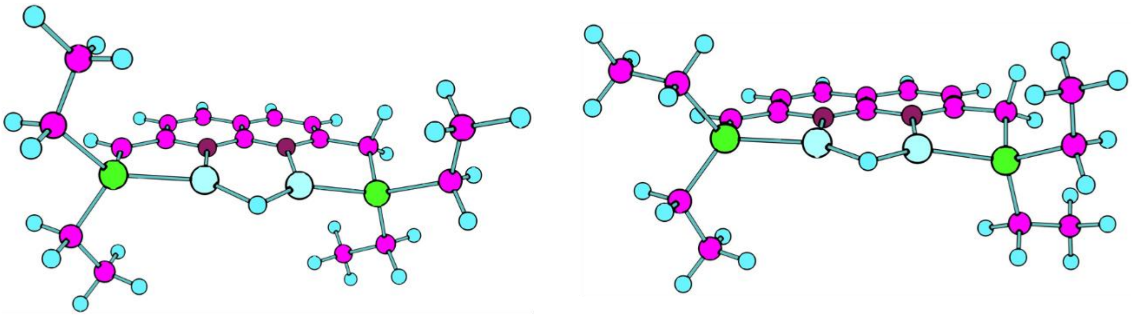

^{Et}PNNP*Cu$_2$H (1) ^{Et}PNNP*Cu$_2$H (2)

Figure S19: Different conformations of the Et groups on ^{Et}(PNNP)Cu$_2$H complexes used to calculate their dimerization equilibrium.*

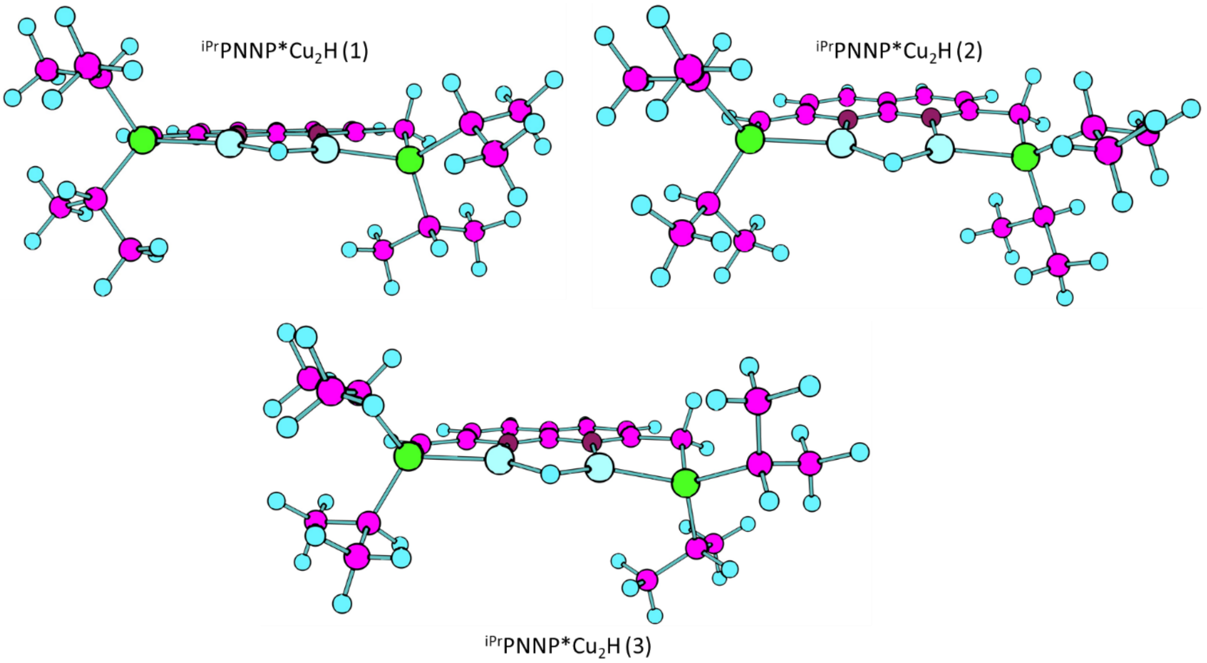

^{iPr}PNNP*Cu$_2$H (3)

Figure S20: Different conformations of the iPr groups on ^{iPr}(PNNP)Cu$_2$H complexes used to calculate their dimerization equilibrium.*

References:

(1) Software update: the ORCA program system, version 4.0” Wiley Interdisciplinary Reviews: Computational Molecular Science, 2017, Vol. 8, Issue 1, p. e1327

(2) Neese, F. “The ORCA program system” Wiley Interdisciplinary Reviews: Computational Molecular Science, 2012, Vol. 2, Issue 1, Pages 73–78.

(3) Stoychev, G. L.; Auer, A. A.; Neese, F. Automatic Generation of Auxiliary Basis Sets. *J. Chem. Theory Comput.* **2017**, *13* (2), 554–562. https://doi.org/10.1021/acs.jctc.6b01041.

(4) Becke, A. D. Density-Functional Exchange-Energy Approximation with Correct Asymptotic Behavior. *Phys. Rev. A* **1988**, *38* (6), 3098–3100. https://doi.org/10.1103/PhysRevA.38.3098.

(5) Perdew, J. P. Density-Functional Approximation for the Correlation Energy of the Inhomogeneous Electron Gas. *Phys. Rev. B* **1986**, *33* (12), 8822–8824. https://doi.org/10.1103/PhysRevB.33.8822.

(6) Weigend, F.; Ahlrichs, R. Balanced Basis Sets of Split Valence, Triple Zeta Valence and

Quadruple Zeta Valence Quality for H to Rn: Design and Assessment of Accuracy. *Phys. Chem. Chem. Phys.* **2005**, *7* (18), 3297. https://doi.org/10.1039/b508541a.

(7) Grimme, S.; Antony, J.; Ehrlich, S.; Krieg, H. A Consistent and Accurate Ab Initio Parametrization of Density Functional Dispersion Correction (DFT-D) for the 94 Elements H-Pu. *J. Chem. Phys.* **2010**, *132* (15), 154104. https://doi.org/10.1063/1.3382344.

(8) Grimme, S.; Ehrlich, S.; Goerigk, L. Effect of the Damping Function in Dispersion Corrected Density Functional Theory. *J. Comput. Chem.* **2011**, *32* (7), 1456–1465. https://doi.org/10.1002/jcc.21759.

(9) Falivene, L.; Cao, Z.; Petta, A.; Serra, L.; Poater, A.; Oliva, R.; Scarano, V.; Cavallo, L. Towards the Online Computer-Aided Design of Catalytic Pockets. *Nat. Chem.* **2019**, *11* (10), 872–879. https://doi.org/10.1038/s41557-019-0319-5.

(10) Guzei, I. A.; Wendt, M. Program Solid-G. 2004.

(11) Kounalis, E.; Lutz, M.; Broere, D. L. J. Tuning the Bonding of a µ-Mesityl Ligand on Dicopper(I) through a Proton-Responsive Expanded PNNP Pincer Ligand. *Organometallics* **2020**, *39* (4), 585–592. https://doi.org/10.1021/acs.organomet.9b00829.

(12) Kounalis, E.; Lutz, M.; Broere, D. L. J. Cooperative H 2 Activation on Dicopper(I) Facilitated by Reversible Dearomatization of an “Expanded PNNP Pincer” Ligand. *Chem. – A Eur. J.* **2019**, *25* (58), 13280–13284. https://doi.org/10.1002/chem.201903724.

(13) Scheerder, A. R.; Lutz, M.; Broere, D. L. J. Unexpected Reactivity of a PONNOP “expanded Pincer” Ligand. *Chem. Commun.* **2020**, *56* (59), 8198–8201. https://doi.org/10.1039/d0cc02166k.

(14) Delaney, A. R.; Yu, L. J.; Coote, M. L.; Colebatch, A. L. Synthesis of an Expanded Pincer Ligand and Its Bimetallic Coinage Metal Complexes. *Dalt. Trans.* **2021**, *50* (34), 11909–11917. https://doi.org/10.1039/d1dt01741a.

(15) Chemcraft - graphical software for visualization of quantum chemistry computations. https://www.chemcraftprog.com

(16) Behlen, M. J.; Zhou, Y. Y.; Steiman, T. J.; Pal, S.; Hartline, D. R.; Zeller, M.; Uyeda, C. Dinuclear Oxidative Addition Reactions Using an Isostructural Series of Ni2, Co2, and Fe2 Complexes. *Dalt. Trans.* **2017**, *46* (17), 5493–5497. https://doi.org/10.1039/c6dt04465d.

(17) Ziegler, M. S.; Levine, D. S.; Lakshmi, K. V.; Tilley, T. D. Aryl Group Transfer from Tetraarylborato Anions to an Electrophilic Dicopper(I) Center and Mixed-Valence µ-Aryl Dicopper(I,II) Complexes. *J. Am. Chem. Soc.* **2016**, *138* (20), 6484–6491. https://doi.org/10.1021/jacs.6b00802.

(18) Zhou, Y. Y.; Hartline, D. R.; Steiman, T. J.; Fanwick, P. E.; Uyeda, C. Dinuclear Nickel Complexes in Five States of Oxidation Using a Redox-Active Ligand. *Inorg. Chem.* **2014**, *53* (21), 11770–11777. https://doi.org/10.1021/ic5020785.

(19) Hung, M. U.; Liao, B. S.; Liu, Y. H.; Peng, S. M.; Liu, S. T. Dicopper Complexes Catalyzed Coupling/Cyclization of 2-Bromobenzoic Acids with Amidines Leading to Quinazolinones. *Appl. Organomet. Chem.* **2014**, *28* (9), 661–665. https://doi.org/10.1002/aoc.3177.

(20) Neese, F. Wennmohs, F., Orca version 4.2.1 Manual, **2019**, https://orcaforum.kofo.mpg.de/app.php/dlext/?cat=10
